# TransBorrow: genome-guided transcriptome assembly by borrowing assemblies from different assemblers

**DOI:** 10.1101/gr.257766.119

**Published:** 2020-08

**Authors:** Ting Yu, Zengchao Mu, Zhaoyuan Fang, Xiaoping Liu, Xin Gao, Juntao Liu

**Affiliations:** 1 School of Mathematics and Statistics, Shandong University (Weihai), Weihai 264209, China;; 2 Key Laboratory of Systems Biology, CAS Center for Excellence in Molecular Cell Science, Institute of Biochemistry and Cell Biology, Shanghai Institutes for Biological Sciences, Chinese Academy of Sciences, University of Chinese Academy of Sciences, Shanghai 200031, China;; 3 Computational Bioscience Research Center (CBRC), Computer, Electrical and Mathematical Sciences and Engineering Division, King Abdullah University of Science and Technology (KAUST), Thuwal 23955, Saudi Arabia

## Abstract

RNA-seq technology is widely used in various transcriptomic studies and provides great opportunities to reveal the complex structures of transcriptomes. To effectively analyze RNA-seq data, we introduce a novel transcriptome assembler, TransBorrow, which borrows the assemblies from different assemblers to search for reliable subsequences by building a colored graph from those borrowed assemblies. Then, by seeding reliable subsequences, a newly designed path extension strategy accurately searches for a transcript-representing path cover over each splicing graph. TransBorrow was tested on both simulated and real data sets and showed great superiority over all the compared leading assemblers.

RNA-seq technology is still widely used throughout the world to explore and study the very complex transcriptomic structures of eukaryotes (Marioni et al. 2008; Wang et al. 2009; Wilhelm and Landry 2009; Marguerat and Bähler 2010) because of its high throughput, high accuracy, and low cost. It is a powerful technology that identifies expressed transcripts and measures isoform expression levels at the whole-transcriptome level with unprecedented accuracy (Wang et al. 2009; Wilhelm and Landry 2009; Marguerat and Bähler 2010; Ozsolak and Milos 2011).

Most eukaryotic genes generally produce multiple isoforms because of alternative splicing in eukaryotes. Therefore, one of the most important tasks is to accurately identify all the expressed transcripts for subsequent biological studies. However, transcripts from the same locus can share exons owing to alternative splicing, and different isoforms from the same gene may have highly variable expression abundances, making the transcriptome assembly problem quite challenging. Moreover, RNA-seq runs generate hundreds of millions of short reads (usually 50–300 bp in length) with ∼2% sequencing errors (Metzker 2010; Canzar et al. 2016). Therefore, computationally identifying all the expressed transcripts from the large amounts of short sequencing reads with unknown sequencing errors poses a great challenge.

Currently available transcriptome assemblers are usually categorized into two strategies: genome-guided (or reference-based) and de novo. In general, if a high-quality genome is available for some species, such as humans, genome-guided assemblers such as Scallop (Shao and Kingsford 2017), TransComb (Liu et al. 2016b), StringTie (Pertea et al. 2015), StringTie2 (Kovaka et al. 2019), Cufflinks (Trapnell et al. 2010), Class2 (Song et al. 2016), Scripture (Guttman et al. 2010), IsoInfer (Feng et al. 2011), IsoLasso (Li et al. 2011), iReckon (Mezlini et al. 2013), CEM (Li and Jiang 2012), Traph (Tomescu et al. 2013), and Mitie (Behr et al. 2013) usually first map all RNA-seq reads to the genome using mapping tools such as HISAT (Kim et al. 2015), STAR (Dobin et al. 2013), TopHat (Trapnell et al. 2009), TopHat2 (Kim et al. 2013), SpliceMap (Au et al. 2010), or GSNAP (Wu and Nacu 2010). Then, a graph model, such as a splicing graph or overlap graph, is built based on the mappings, and different methods are implemented to search for transcript-representing paths in the constructed graphs. Different from the reference-based strategy, which benefits from high-quality genomes, the de novo strategy, with assemblers such as TransLiG (Liu et al. 2019), BinPacker (Liu et al. 2016a), Bridger (Chang et al. 2015), Trinity (Grabherr et al. 2011), ABySS (Simpson et al. 2009), SOAPdenovo-Trans (Xie et al. 2014), and IDBA-Tran (Peng et al. 2013), usually builds graph models and assembles all expressed transcripts directly from the RNA-seq reads. Therefore, the de novo strategy is more challenging than the genome-guided strategy, and its assembly accuracy is generally much lower. However, the de novo strategy plays an important role in studying species for which genome assemblies of high quality are not available at the moment.

For genome assembly, a variety of assembly tools are available, but it is not always obvious which tool to use for a specific genome. Therefore, a compelling approach is to merge multiple assemblies to produce a higher-quality consensus assembly (Alhakami et al. 2017). Several tools for merging multiple assemblies have been developed, such as CISA (Lin and Liao 2013), GAA (Yao et al. 2012), GAM_NGS (Vicedomini et al. 2013), GARM (Soto-Jimenez et al. 2014), Metassembler (Wences and Schatz 2015), and MIX (Soueidan et al. 2013) via different merging strategies. For example, the CISA tool first selects representative contigs and discards contained contigs. It then extends representative contigs and detects misassembly in the representative contigs by aligning them to query contigs. Finally, the resulting contigs are iteratively merged. Another tool, MIX, uses an extension graph to determine a set of nonoverlapping maximal independent longest paths to merge contigs. Contigs not included in any path are examined for duplications. Contigs that are contained or nearly contained are removed, and the rest are added to the assembly.

For transcriptome assembly, no assembler consistently generates the most accurate assemblies when tested across different RNA-seq data sets, and it is difficult to determine which assembler to use for a specific RNA-seq data set (Smith-Unna et al. 2016; Voshall and Moriyama 2018). Therefore, it is imperative to develop approaches to merge assemblies from different assemblers (Haas et al. 2003; Venturini et al. 2018). The de novo assemblers EvidentialGene (Gilbert 2013) and Concatenation (Cerveau and Jackson 2016) try to address the limitations of individual assemblers, ideally keeping the correctly assembled transcripts. Both tools process the transcripts obtained from multiple assemblers by clustering the transcripts and predicting coding regions to determine the representative sequence for each cluster. The de novo assembler Trans-ABySS (Robertson et al. 2010) attempts to solve this problem by merging assemblies from ABySS (Simpson et al. 2009) using different *k*-mer lengths. The genome-guided assembler Mikado (Venturini et al. 2018) generates a coherent transcript annotation by integrating multiple RNA-seq assemblies from multiple samples. It defines loci, scores transcripts, determines a representative transcript for each locus, and finally returns a set of gene models. In addition, some other assembling tools for reconstructing a consensus transcriptome from multiple RNA-seq samples have also been developed, such as TACO (Niknafs et al. 2017) and StringTie-merge (Pertea et al. 2015).

In this study, we developed a new genome-guided assembler TransBorrow, which assembles transcripts by first building splicing graphs based on the mapped reads and extracting reliable paired subpaths from splicing graphs. It then borrows reliable subsequences from different assemblies by building a so-called colored graph. Then, those reliable subsequences and paired subpaths are mapped to the splicing graphs as reliable subpaths for guiding the correct assemblies of expressed transcripts. Finally, a newly designed path extension method is applied to search for a transcript-representing path cover over each splicing graph by seeding those reliable subpaths (for the flowchart of TransBorrow, see Fig. 1). Below, we describe the algorithmic approaches used in TransBorrow in detail and benchmark it against the state-of-the-art transcriptome assemblers StringTie2, Scallop, and Cufflinks and two merging-based assemblers StringTie-merge and TACO on both simulated and real RNA-seq data sets.

**Figure 1.**
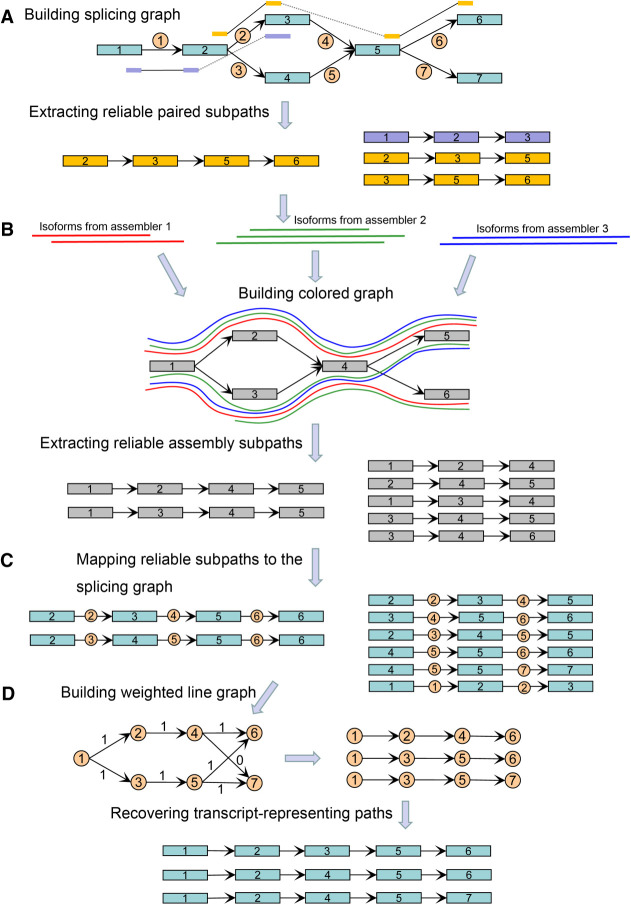
Flowchart of the TransBorrow algorithm. (*A*) Building splicing graph and extracting reliable paired subpaths; (*B*) building colored graph and extracting reliable assembly subpaths; (*C*) mapping reliable subpaths to the splicing graph; (*D*) recovering transcript-representing paths.

## Results

TransBorrow is a transcriptome assembler that takes advantage of assemblies from different assembly tools by searching for reliable assembly subpaths from different assemblies and then seeding these subpaths for transcript-representing path extensions subsequently in each splicing graph. To evaluate its performance, we first assembled the RNA-seq reads by using different assemblers, and then the algorithm TransBorrow was run by merging these different assemblies. The results showed that TransBorrow effectively takes advantage of the assemblies from different tools and that TransBorrow has enhanced performance compared with that of other assembly tools.

In this study, the latest alignment tools HISAT2 and STAR were used for mapping the RNA-seq reads to a reference genome, and then the leading assemblers Scallop, StringTie2, and Cufflinks were applied to assemble all the expressed transcripts of both simulated and real data. Then, we ran TransBorrow by merging the assemblies from the three assemblers and comparing its performance with each of them. In addition, two merging-based assemblers, StringTie-merge and TACO, were also tested, which combined assemblies from different samples (rather than assemblies from different assemblers) to improve assembly accuracy. In this study, we also compared with StringTie-merge and TACO by taking the assemblies from StringTie2, Scallop, and Cufflinks (as those are from different samples). The detailed commands for running all the mappers and assemblers and the versions of all the tools are described in the Supplemental Material. The common comparison criteria used in this study were that a reference transcript is considered to be correctly detected if and only if its intron chain is exactly matched with an assembled transcript. The human genome GRCh37/hg19 and all the reference transcripts downloaded from the UCSC hg19 gene annotation were used as a reference genome and transcriptome, respectively.

### Performance of TransBorrow on simulated data

The FLUX simulator (Griebel et al. 2012) was used to generate the simulation data (150-bp length, approximately 73 million paired-end reads), on which we tested the performance of TransBorrow, Scallop, StringTie2, and Cufflinks by commonly used criteria such as assembly accuracy (recall and precision) at both the transcript and gene levels and the identification of transcripts with different expression levels (low, medium, and high). The parameters and running commands for generating the simulated data set are described in the Supplemental Material.

#### Comparison of assembly accuracy at the transcript and gene levels

We first ran Scallop, StringTie2, and Cufflinks on the simulated data by using the mapping results from both HISAT2 and STAR. Then TransBorrow was run by merging the assemblies from the three assemblers on the mapping results from HISAT2 and STAR. The accuracy was evaluated by recall (the fraction of correctly detected expressed transcripts out of all the expressed transcripts) and precision (the percentage of assembled transcripts that exactly matched an expressed transcript).

**Figure 2.**
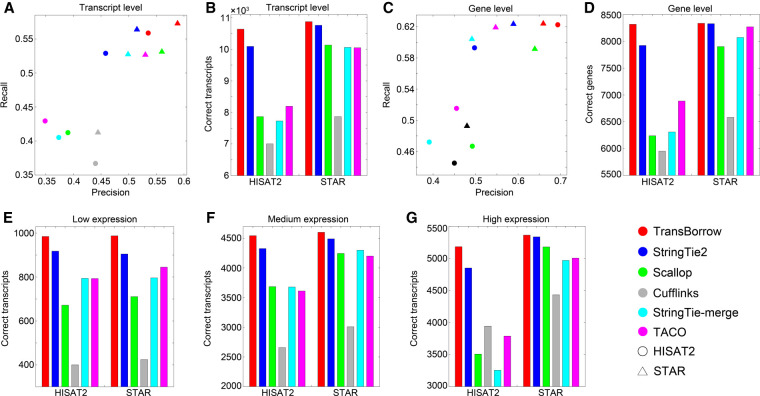
Performance comparisons of the assemblers on the simulated data. (*A*) Comparisons of assembly accuracy of the assemblers at the transcript level. (*B*) The number of correctly assembled transcripts by the assemblers. (*C*) Comparisons of assembly accuracy of the assemblers at the gene level. (*D*) The number of correctly detected genes by the assemblers. (*E*–*G*) Comparisons of detected transcripts with low, medium, or high expression levels on the simulated data.

Based on both HISAT2 and STAR mapping, TransBorrow consistently achieved the highest recall and precision among all the compared assemblers on the simulated data (see Fig. 2A; for details, see Supplemental Table S1). For the correctly assembled transcripts based on HISAT2 and STAR mapping, TransBorrow correctly detected 5.64% and 1.29% more expressed transcripts than StringTie2, 35.58% and 7.53% more than Scallop, 52.29% and 38.55% more than Cufflinks, 37.96% and 8.3% more than StringTie-merge, and 30.13% and 8.44% more than TACO (see Fig. 2B; for details, see Supplemental Table S1). Therefore, TransBorrow performed better than all the other compared assemblers on the simulated data at the transcript level.

We further compared the performance of the assemblers in identifying expressed genes. A gene is considered to be correctly detected if at least one of its isoforms is correctly assembled. Similarly, recall (at the gene level) is defined as the fraction of correctly detected genes out of the expressed genes, and precision (at the gene level) is defined as the fraction of correctly detected genes out of all assembled genes.

After running the assemblers based on both HISAT2 and STAR mapping, the recall and precision of TransBorrow again achieved the highest among all the compared assemblers (see Fig. 2C; for details, see Supplemental Table S1). Regarding the correctly detected genes, TransBorrow correctly detected 4.96% more genes than StringTie2 based on HISAT2 mapping, 33.33% and 5.48% more than Scallop based on HISAT2 and STAR mapping, 39.79% and 26.65% more than Cufflinks, 31.83% and 3.26% more than StringTie-merge, and 20.78% and 0.8% more than TACO (see Fig. 2D; for details, see Supplemental Table S1). Therefore, TransBorrow reached the best performance among all the compared assemblers under both HISAT2 and STAR mappings at the gene level.

#### Comparison of detected transcripts with different expression levels

In theory, splicing isoforms with relatively lower expression levels are more difficult to correctly assemble than those with higher expression levels (Canzar et al. 2016; Shao and Kingsford 2017). To compare the performance of the assemblers in identifying transcripts with different expression levels, especially those with relatively lower expression levels, as previously described (Shao and Kingsford 2017), the expressed transcripts of the simulated data were first equally divided into three parts according to their expression levels, which corresponded to lowly, moderately, and highly expressed transcripts.

After comparison, the results showed that TransBorrow correctly detected the largest number of expressed transcripts, regardless of expression levels, based on both HISAT2 and STAR mappings (for details, see Fig. 2E–G). More importantly, TransBorrow correctly detected 7.3% and 9.17% more lowly expressed transcripts than StringTie2, 46.58% and 38.96% more than Scallop, 146.25% and 133.02% more than Cufflinks, 24.06% and 24.12% more than StringTie-merge, and 24.21% and 16.92% more than TACO, which clearly showed that TransBorrow performed the best in identifying transcripts with different expression levels, especially those with low expression levels.

### Performance of TransBorrow on real data

The advantage of simulated data is that its ground truth is known; however, it is not able to capture all the features of real RNA-seq data. Therefore, performance evaluations of the assemblers should also be implemented on real data. As the ground truth of real data is difficult to know, we set all the reference transcripts downloaded from the UCSC gene annotation hg19 as the ground truth in this study. Four real data sets—including R1, K562 cells (replicate 1); R2, K562 cells (replicate 2); R3, H1 cells (replicate 1); and R4, H1 cells (replicate 2)—were collected from the NCBI Sequence Read Archive (SRA) with the accession codes SRR387661, SRR387662, SRR307911, and SRR307912, respectively. The four data sets, R1, R2, R3, and R4, contain approximately 125 million, 88 million, 41 million, and 37 million paired-end reads, respectively. Then, the performance of the assemblers was evaluated on the four real data sets by using the same criteria as that used for the simulated data.

#### Comparison of assembly accuracy at the transcript level

**Figure 3.**
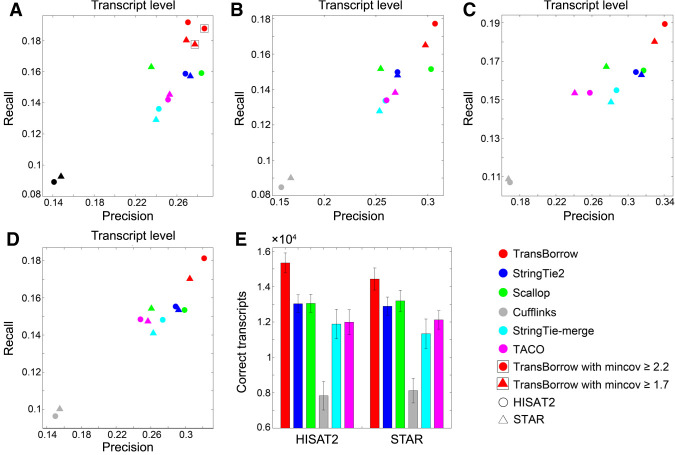
Accuracy comparisons of the assemblers on the four real data sets at the transcript level. (*A*–*D*) Comparisons of assembly accuracy of the assemblers on data sets R1, R2, R3, and R4, respectively. (*E*) The average number of correctly assembled transcripts by the assemblers on data sets R1, R2, R3, and R4.

After running all the assemblers on the four real data sets, the results showed that TransBorrow achieved the highest recall on all four data sets based on both HISAT2 and STAR mappings (see Fig. 3A–D; for details, see Supplemental Table S2). In terms of precision, TransBorrow reached the highest precision among all compared assemblers on the data sets R2, R3, and R4 based on both HISAT2 and STAR mappings (see Fig. 3A–D; for details, see Supplemental Table S2). On the first data set R1, the precision of TransBorrow is slightly lower than that of Scallop based on HISAT2 mapping and slightly lower than that of StringTie2 based on STAR mapping. However, the *F*-score of TransBorrow is the highest (for details, see Supplemental Fig. S9), which indicates that the overall performance of TransBorrow was better than those of both StringTie2 and Scallop with their default settings. By default, assemblers filtered their assembled transcripts with low estimated expression levels after transcriptome assembly. Therefore, after filtering, the recall will generally decline, whereas the precision will increase. This flexible filtering parameter corresponds to a trade-off between recall and precision. If the users adjust the parameter, TransBorrow could reach both higher recall and precision than StringTie2 and Scallop. For example, if we filtered the assembled transcripts of TransBorrow by using parameters 2.2 and 1.7 under HISAT2 and STAR mappings to make its precision slightly higher than that of Scallop and StringTie2 on data set R1, and we found that the recall and precision of TransBorrow were 18.78% and 28.66% under HISAT2 mapping, and 17.76% and 27.74% under STAR mapping, which indicates that TransBorrow showed both higher recall and precision than Scallop and StringTie2 (see Fig. 3A; for details, see Supplemental Table S2). In addition, the recall/precision curves give more comprehensive comparison between different methods (for details, see Supplemental Fig. S14).

For the correctly assembled transcripts based on HISAT2 mappings, TransBorrow correctly detected 15.19%–20.82% more transcripts than StringTie2, 14.61%–20.52% more than Scallop, 76.81%–114.93% more than Cufflinks, 22.2%–40.84% more than StringTie-merge, and 22.12%–34.95% more than TACO on the four real data sets (see Fig. 3E; for details, see Supplemental Table S2). Based on STAR mappings, TransBorrow correctly detected 10.59%–14.7% more transcripts than StringTie2, 7.78%–10.54% more than Scallop, 65.62%–94.52% more than Cufflinks, 20.73%–39.58% more than StringTie-merge, and 15.5%–24.05% more than TACO on the four real data sets (see Fig. 3E; for details, see Supplemental Table S2). Therefore, TransBorrow performed better than all the other compared assemblers on the four real data sets at the transcript level.

#### Comparison of assembly accuracy at the gene level

**Figure 4.**
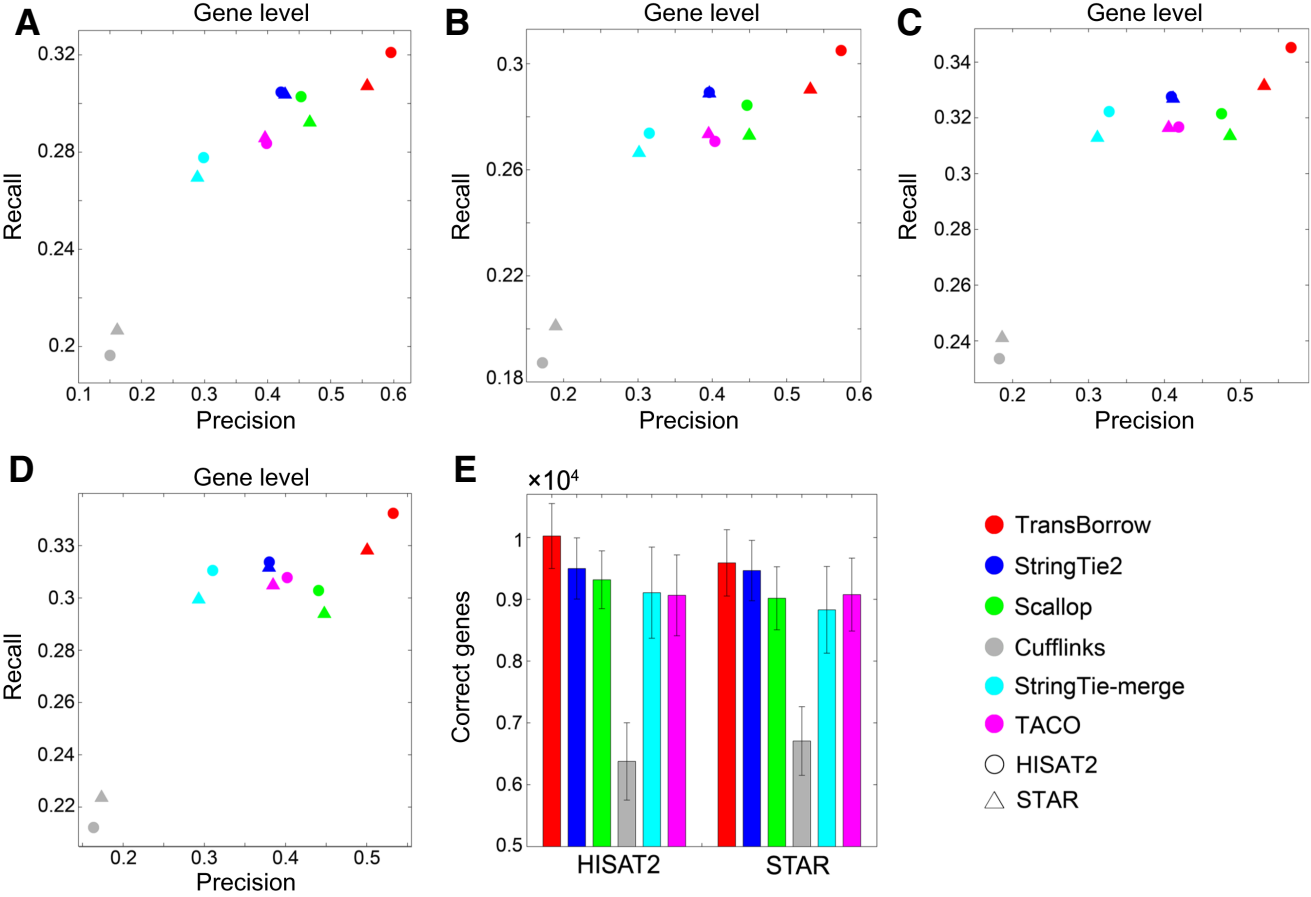
Accuracy comparisons of the assemblers on the four real data sets at the gene level. (*A*–*D*) Comparisons of assembly accuracy of the assemblers on data sets R1, R2, R3, and R4, respectively. (*E*) The average number of correctly detected genes by the assemblers on data sets R1, R2, R3, and R4.

We then compared the accuracy of the assemblers at the gene level using the four real data sets. After running the assemblers based on both HISAT2 and STAR mappings, TransBorrow reached much higher recall and precision than any of the compared assemblers on all four real data sets (see Fig. 4A–D; for details, see Supplemental Table S3).

Regarding the correctly detected genes based on the HISAT2 mappings, TransBorrow correctly detected 5.35%–5.94% more genes than StringTie2, 6%–9.74% more than Scallop, 47.76%–63.49% more than Cufflinks, 7.04%–15.57% more than StringTie-merge, and 7.97%–13.2% more than TACO (see Fig. 4E; for details, see Supplemental Table S3). Based on STAR mappings, TransBorrow correctly detected 0.52%–2.12% more genes than StringTie2, 5.11%–8.26% more than Scallop, 37.53%–48.65% more than Cufflinks, 5.94%–13.96% more than StringTie-merge, and 4.37%–7.5% more than TACO on the four real data sets (see Fig. 4E; for details, see Supplemental Table S3). Therefore, TransBorrow performed better than all the other compared assemblers on the four real data sets at the gene level.

#### Comparison of identifying transcripts with different expression levels

For real data, the exact expression abundances of the transcripts were unknown to us. To make relatively fair comparisons of the assemblers in identifying transcripts with different expression levels on real data, we first estimated the expression levels (TPM values) of the whole-reference transcripts using the well-known abundance estimator kallisto (Bray et al. 2016), based on which the reference transcripts could be equally divided into three parts corresponding to the transcripts with low, medium, and high expression levels as we did on the simulated data.

**Figure 5.**
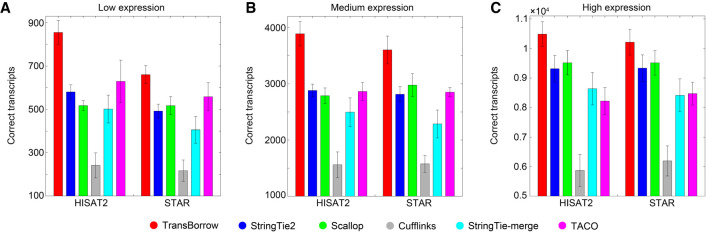
Performance comparisons of the assemblers in identifying transcripts with different expression levels on the real data. (*A*–*C*) The average number of correctly assembled transcripts with different expression levels by the assemblers on data sets R1, R2, R3, and R4.

In comparison with any of the other assemblers, TransBorrow correctly detected more reference transcripts, regardless of expression levels, for all four real data sets based on both HISAT2 and STAR mappings (for details, see Fig. 5A–C). It is worth mentioning that based on HISAT2 mappings, TransBorrow correctly detected 44.19%–54.53% more lowly expressed transcripts from the four data sets than StringTie2, 51.66%–79.37% more than Scallop, 191.72%–361.22% more than Cufflinks, 52.69%–93.16% more than StringTie-merge, and 21.18%–59.15% more than TACO. For the STAR mappings, TransBorrow correctly detected 31.91%–36.22% more lowly expressed transcripts from the four data sets than StringTie2, 21.81%–37.8% more than Scallop, 161.78%–312.8% more than Cufflinks, 47.39%–84.97% more than StringTie-merge, and 11%–28.71% more than TACO. Therefore, the comparison showed that TransBorrow consistently maintained considerably superior performance in identifying lowly expressed transcripts not only on simulated data but also on real data sets.

#### Comparison of running time and memory usage

To compare the running time and memory usage of the assemblers, all the assemblers were run on the same server with 96 GB of memory and a 12-core CPU. The results showed that Scallop and StringTie2 ran the fastest on all the four real data sets. TransBorrow ran slightly slower than Scallop and StringTie2, but several times faster than Cufflinks. For example, on data sets R2 (88 million reads) and R3 (41 million reads) under STAR mappings, the running time of StringTie2 was 10 and 6 min, respectively. The running time of Scallop was 24 and 9 min, respectively, and the running time of TransBorrow was 45 and 33 min, respectively. However, the running time of Cufflinks was 148 and 97 min, respectively. For memory usage, the memory cost by StringTie2 was ∼1 GB on all the four data sets, and all the other assemblers showed a similar trend, with maximum memory usage of no more than 10 GB in most cases (for the running time and memory usage of all the four real data sets, see Supplemental Table S5). Overall, although TransBorrow is not the most efficient in running time and memory usage, it is quite acceptable for practical use. The merging-based approaches, StringTie-merge and TACO, are different from the above assemblers because they only take the assemblies from the other assemblers as their inputs. Their running time was much faster, and their memory usage was also less than that of all the above assemblers (for details, see Supplemental Table S5).

### Additional evaluations

In addition to the evaluations presented above, the Supplemental Material also includes evaluating the assembly accuracy of all assemblers on an additional 101 RNA-seq samples from the species *Homo sapiens*, *Saccharomyces cerevisiae*, *Drosophila melanogaster*, *Caenorhabditis elegans*, *Mus musculus*, *Arabidopsis thaliana*, and *Zea mays* (see Supplemental Figs. S1–S4). The assembly accuracy of the assemblers by using spike-in and single-cell RNA-seq data sets (Supplemental Figs. S5–S7), the performance of the assemblers at identifying long noncoding transcripts (see Supplemental Fig. S8) and the *F*-score of the assemblers on all the data sets (see Supplemental Figs. S9–S12) were also evaluated in the Supplemental Material. Moreover, some other evaluations were also performed, including the comparison of the performance of TransBorrow (by using the assemblies from StringTie, Scallop, and Cufflinks) with that of StringTie2 (see Supplemental Fig. S13), the recall/precision curves of each assembler on all the data sets (see Supplemental Figs. S14–S17), the comparison of the performance of TransBorrow with an approach that simply combined the assembled transcripts from different assemblers (see Supplemental Fig. S18), and the assembled transcripts of a gene with complicated splice junction patterns in the form of a Genome Browser snapshot (for details, see Supplemental Fig. S19).

## Discussion

In this study, we present a novel genome-guided assembler, TransBorrow, for transcriptome assembly using short RNA-seq reads. Compared with three leading assemblers of the same kind on both simulated and real data sets, TransBorrow consistently performs the best under commonly used criteria. The superiority of TransBorrow may be attributed to the following.

First, TransBorrow attempts to identify all expressed transcripts by taking advantage of different assemblies from other assemblers. The reliable subsequences generated in this step serve as seeds and effectively guide the subsequent assembly process. Second, TransBorrow develops a new graph model, the colored graph, which was built by merging different assemblies. Based on colored graphs, reliable subsequences could be accurately and efficiently extracted from merged assemblies. Third, TransBorrow constructs a weighted line graph for each splicing graph, whose edge weight exactly indicates the correct connections between the incoming and outgoing edges for each node of the splicing graph. Fourth, TransBorrow implements a newly designed path extension strategy for searching for a transcript-representing path cover over each weighted line graph by seeding the extracted reliable subpaths and iteratively choosing the best neighbor for extension.

Although we have seen great advantages of TransBorrow, further improvements could still be made for TransBorrow in the future. For example, the current version of TransBorrow is not compatible with long-read RNA-seq data sets (e.g., Pacific Biosciences [PacBio] or Oxford Nanopore Technologies [ONT]). Similar to other assemblers, StringTie2, Scallop, and Cufflinks, the current version of TransBorrow performs transcriptome assembly in each individual gene locus without considering the resolution of chimeric transcripts. And the current version of TransBorrow is a genome-guided assembler, which is not compatible with de novo assemblies. The future version of TransBorrow will attempt to solve these problems and make further improvements. For the development of TransBorrow, difficulties existed when we were building it. For example, different assemblers used by TransBorrow may generate quite different exon–intron boundaries for some genes, which makes the colored graphs very complicated. In terms of the efficiency of TransBorrow, it depends in part on the performance of the borrowed assemblies, and we tried to optimize the performance of the assemblers that were used upstream of TransBorrow by adjusting their parameters, such as minimum isoform abundance, minimum transcript length, and minimum junction coverage, and the performance of TransBorrow was only slightly affected by these operations. Therefore, running the assemblers upstream of TransBorrow by using their default parameters would be good. In addition, we tried to replace Cufflinks with two other assemblers, CLASS2 and Strawberry, and tested the performance of TransBorrow. We found that the performance of TransBorrow was not improved when replacing Cufflinks with CLASS2 or Strawberry. For example, on the simulated data set, the recall and precision of TransBorrow by using StringTie2, Scallop, and Cufflinks were 57.12% and 60.32% versus 57.52% and 55.07% when replacing Cufflinks with CLASS2 and 57.14% and 55.57% when replacing Cufflinks with Strawberry. Therefore, we ran TransBorrow by using the assemblies from Cufflinks. Moreover, we also tested TransBorrow on four single-cell RNA-seq data sets, and it also showed appreciable improvements over the applied assemblers (see Supplemental Material; Supplemental Fig. S7).

Tools such as EvidentialGene, Concatenation, and Mikado also perform assemblies by combining assemblies from different assemblers, which is similar to TransBorrow. Different from the three tools, TransBorrow performs transcriptome assembly from the original read mapping results by building splicing graphs and searching for path covers over splicing graphs, and those combined assemblies from different assemblers effectively provide reliable subpaths for TransBorrow guiding its accurate assembly. However, the three tools EvidentialGene, Concatenation, and Mikado perform assemblies completely based on the results from other assemblers, which clearly limits their performance in identifying novel transcripts. In addition, the performance of Mikado also relied on additional reference information, such as the BLAST information, whereas the current version of TransBorrow does not make use of additional reference information. The two assemblers EvidentialGene and Concatenation were designed for processing combined assemblies in the FASTA format, different from TransBorrow and Mikado, which use assemblies in the GTF format.

To the best of our knowledge, TransBorrow is the first genome-guided transcriptome assembler that uses assemblies from different tools by searching for reliable assembly subpaths from different assemblies and then seeding these subpaths for transcript-representing path extensions in each splicing graph. The software has been developed to be user-friendly and is expected to play a crucial role in new discoveries of transcriptome studies using RNA-seq, especially in complicated human diseases related to abnormal splicing events and expression levels, such as cancers.

## Methods

### Building splicing graphs and extracting reliable paired subpaths

Assembly of expressed transcripts in this study is completed with the traditional graph model, the splicing graph. Therefore, we first build accurate splicing graphs and then collect all subpaths in the graphs supported by the paired-end sequencing reads.

#### Building splicing graphs

The splicing graph is constructed from mapping the RNA-seq reads to a reference genome using mapping tools such as HISAT2 or STAR. According to the mapping results, reads are usually clustered into corresponding gene loci, and a splicing graph is generally built for each gene. The exon–intron boundaries and exon–exon junctions are derived from those mapping reads spanning two or more exons. Generally, each node in a splicing graph represents an exon in the corresponding gene, and a directed edge between two nodes means a splicing junction between the two exons. For a splicing graph, we assign a weight to each edge depending on the number of reads spanning it.

Theoretically, edges in the splicing graphs can capture most splicing events in the expressed transcripts, and the sequencing depth information is appropriately integrated into the graph as the edge weight. Then the task of transcript assembly is to accurately search for an edge-path cover for the splicing graph, each path of which represents an expressed transcript.

#### Extracting reliable paired subpaths

To make full use of the paired-end information in the subsequent assembly procedure, we search for all the subpaths in each splicing graph supported by the paired-end reads, named paired subpaths.

In detail, for each two paired-end reads *r*_1_ and *r*_2_, if *r*_1_ spans a path *P*_1_ = *n*_*i*1_ → *n*_*i*2_ → … → *n*_*ip*_ in a splicing graph *G* while *r*_2_ spans *P*_2_ = *n*_*j*1_ → *n*_*j*2_ → … → *n*_*jq*_, we will search for all paths from *n*_*ip*_ to *n*_*j1*_ in graph *G*. If there exists one and only one path *P*_*in*_ = *n*_*ip*_ → *n*_*m*1_ → *n*_*m*2_ → … → *n*_*ms*_ → *n*_j1_ between *n*_*ip*_ and *n*_*j1*_ satisfying *p* + *s* + *q* ≥ 3, then reads *r*_*1*_ and *r*_*2*_ are connected by the path *P*_*in*_, and the corresponding paired subpath is extracted as *P* = *P*_1_ → *P*_*in*_ → *P*_2_ (see Fig. 1A). After all the paired-end reads in graph *G* are processed, we obtain a set *S*_*p*_ of all paired subpaths. Different paired-end reads may generate the same paired path. Therefore, we record the number of paired-end reads that generate each paired subpath *P,* and this number is called the coverage of path *P*.

In fact, some paired subpaths may be erroneously extracted because of mapping or sequencing errors. These paired subpaths usually show very low coverage and need to be removed. Although a paired subpath *P* is erroneously extracted, the subpaths of *P* may be reliable and should not be removed. Therefore, we attempt to obtain all reliable paired subpaths by the following steps. Given a paired subpath *P*, we first extract all the subpaths of length three (called paired 3-subpaths) by decomposing the paired subpath *P*. For example, a paired subpath *P* = *n*_1_ → *n*_2_ → *n*_3_ → *n*_4_ will be decomposed into two paired 3-subpaths, *P*_1_ = *n*_1_ → *n*_2_ → *n*_3_ and *P*_2_ = *n*_2_ → *n*_3_ → *n*_4_. Different paired subpaths usually have different coverage, whereas they may generate the same paired 3-subpath. Therefore, for each paired 3-subpath, we record the sum of coverage of all paired subpaths that generate the paired 3-subpath, and this number is called the coverage of the paired 3-subpath. A paired 3-subpath is defined as a reliable paired 3-subpath if its coverage is no less than two, and all reliable paired 3-subpaths can be sorted by their coverage. Similarly, we could extract all reliable paired 4-subpaths, paired 5-subpaths, …, and paired *n*-subpaths, where *n* is the length of the longest paired subpath (see Fig. 1A). Finally, we can obtain all reliable paired subpaths of different lengths, cluster them according to their lengths, and sort them in each cluster by their coverage.

### Building colored graphs and extracting reliable assembly sequences

The main contribution of TransBorrow is to take advantage of the assemblies from different assemblers, which is achieved by extracting all reliable sequences of the assembled transcripts from different assembly tools. These extracted reliable sequences together with the above reliable paired subpaths then serve as key information guiding the subsequent assembly procedure.

#### Building colored graphs

To accurately search for all reliable subsequences, we first build a novel graph model, the colored graph, as follows. Given the merged transcripts assembled by two or more different assemblers, we first cluster all the transcripts into different gene loci. For each gene locus, a colored graph *G*_*c*_ is constructed with nodes and edges representing the exons and splice junctions appearing in the merged transcripts. Then each of the merged transcripts corresponds to a unique path in the colored graph, named an assembled-transcript-representing path. As each graph *G*_*c*_ is built from those assembled-transcript-representing paths and the paths belong to different assemblers, we call graph *G*_*c*_ a colored graph (see Fig. 1B). Based on colored graphs, reliable sequences of the merged transcripts can be effectively extracted as follows.

#### Extracting reliable assembly subpaths in colored graphs

The merged transcripts were predicted by different assemblers; therefore, the merged transcripts usually contain more true positives than the single assembly by any of the assemblers. However, the false positives would also be much more than each single assembly. To extract reliable sequences from the merged transcripts, we take the following steps. It is worth mentioning that a reliable sequence means a segment or the whole of an assembled transcript, which corresponds to a unique subpath of a colored graph.

In theory, if a subpath of a colored graph is covered by a transcript assembled by only one assembler, then this subpath has very low reliability. However, if a subpath is detected by two or more assemblers, then it should have relatively higher reliability. To obtain reliable assembly subpaths based on such considerations, we first extract all the subpaths of length three (called assembly 3-subpaths) by decomposing each assembled-transcript-representing path in the colored graph, similar to that in the subsection “Extracting reliable paired subpaths.” Different assemblers may generate the same assembly 3-subpath; we record the number of assemblers that generate the assembly 3-subpath, and the number is named the depth of the assembly 3-subpath. An assembly 3-subpath is defined as a reliable assembly 3-subpath if its depth is no less than two, and all reliable assembly 3-subpaths can be sorted by their depth. Similarly, we could extract all the reliable assembly 4-subpaths, assembly 5-subpaths, …, and assembly *m*-subpaths, where *m* is the length of the longest assembled-transcript-representing path (see Fig. 1B). Finally, we can obtain all reliable assembly subpaths of different lengths, cluster them according to their lengths, and sort them in each cluster by their depth.

### Mapping reliable subpaths to the splicing graphs

The assembly procedure is performed on splicing graphs, and all reliable paired subpaths and assembly subpaths actually guide the assembly process on splicing graphs. Therefore, we need to map all the reliable assembly subpaths to splicing graphs; then each reliable assembly subpath corresponds to a unique subpath of a splicing graph. To effectively map those assembly subpaths to the splicing graphs, we first build a hash table recording all splicing graphs. For each edge in a splicing graph, the key of the hash table records its corresponding splicing junction positions on a specific chromosome, and the value of the hash table records its graph and edge indexes. Then each assembly subpath could be effectively located to a splicing graph according to the splice junctions in the assembly subpath.

After mapping all the reliable assembly subpaths to splicing graphs, we combine the assembly subpaths and paired subpaths and remove the redundant subpaths (for the redundant subpaths appearing both in the set of assembly subpaths and paired subpaths, we keep only one copy), and the combined subpaths are called reliable subpaths (see Fig. 1C). These reliable subpaths will serve as the seeds and guide the subsequent transcript assembly.

### Searching for transcript-representing paths by seeding reliable paths

Theoretically, each reliable subpath corresponds to a segment of an expressed transcript and therefore should be covered by at least one transcript to be assembled. To achieve this goal, we first create a weighted line graph for each splicing graph, and then a transcript-representing path cover over each line graph is obtained by a newly designed path extension strategy.

#### Building the weighted line graph

To accurately connect the incoming and outgoing edges of each node in a splicing graph *G*, we first build a line graph *L*(*G*) of the splicing graph *G* with nodes representing the edges in *G* and an edge representing two incident edges in *G*. The weight of each node in *L*(*G*) is defined by the coverage of the corresponding edge in *G*, whereas each edge is weighted by solving a quadratic program updated from our previous study (Liu et al. 2019). The quadratic program effectively integrates the information from transcript coverage differences and extracts reliable subpaths (for details, see Supplemental Material). Then, the edge coverage in *L*(*G*) clearly indicates the correct connections between incoming and outgoing edges of each node in graph *G*, with an edge (*e*_*i*_, *e*_*j*_) weight in *L*(*G*) of 1, meaning that this edge comes from an expressed transcript with a high probability and zero otherwise (see Fig. 1D).

#### Searching for transcripts by a novel path extension technique

After assigning weights to both the nodes and edges of the weighted line graph *L*(*G*), a newly designed path extension strategy was applied to assemble all the expressed transcripts by searching for an optimal transcript-representing path cover over the weighted line graph. Before processing path extension, all the reliable subpaths of the splicing graphs should correspond to the paths of the line graphs, and a reliable *n*-subpath in a splicing graph should correspond to a unique reliable (*n* − 1)-subpath in the corresponding line graph. Then TransBorrow searches for all the expressed transcripts for a line graph *L*(*G*) by the following steps.

**Step 1.** Choose the longest reliable subpath *p_r_ = p*_r1_ → *p*_*r2*_ → … → *p*_*rm*_ that is not covered by any predicted path (or choose an unused node in *L*(*G*) if all the reliable subpaths have been included in the predicted paths) as a seed and extend it to one of its right neighbors, with the edge weight being one. If there are multiple choices, we choose the neighbor *n*_*i*_, which is supported by a reliable 2-subpath *p*_*rm*_ → *n*_*i*_. If there are still multiple choices, we choose the neighbor *n*_*j*_, which is supported by a reliable 3-subpath *p*_*r*(*m−1*)_ → *p*_*rm*_ → *n_j_.* The process is iterated until no neighbor is supported by any longer reliable subpath. If there are still multiple choices, then the neighbor with the largest node weight is selected for extension. The extension is continued until the last node of the current path has no outgoing edges. A similar process could be performed for left extensions, and then a transcript-representing path *p*_*t*_ is predicted.
**Step 2.** Define *c*_*min*_ as the minimum node weight in the extended path *p*_*t*_, and then we update each node weight *c*(*n*) of the line graph *L*(*G*) to be *c*(*n*)-*c*_*min*_ if node *n* is included in the path *p*_*t*_.

The above extension process is repeated until all the reliable subpaths and nodes in the line graph *L*(*G*) have been covered by the predicted paths, and then a transcript-representing path cover over the line graph is obtained, each path of which corresponds to a unique path in the splicing graph *G*.

### Software availability

The source code for the latest version of TransBorrow package is available at https://sourceforge.net/projects/transcriptomeassembly/ files/TransBorrow/ and as Supplemental Code.

## Data access

The simulated data set used in this study is available at https:// sourceforge.net/projects/transcriptomeassembly/files/TransBorrow/Data/. All the real data sets were downloaded from NCBI SRA with the accession codes recorded in Supplemental Table S4. The reference genome and transcripts used for evaluating the performance of the assemblers are described in Supplemental Material.

## Competing interest statement

The authors declare no competing interests.

## Supplementary Material

Supplemental Material
