# Supplementary material for: TransBorrow: genome-guided transcriptome assembly by borrowing assemblies from different assemblers
Source: Genome Res. 2020 Aug;30(8):1181–90. doi: 10.1101/gr.257766.119 (PMC7462071; doi:10.1101/gr.257766.119)
Supplement: Supplemental Material [file supp_30_8_1181__index.html]

TransBorrow: genome-guided transcriptome assembly by borrowing assemblies from different assemblers — Supplemental Material 

# TransBorrow: genome-guided transcriptome assembly by borrowing assemblies from different assemblers

## Supplemental Material

- Supplemental\_Material\_.pdf
- Supplemental\_Code.zip
- Supplemental\_Table\_S1.xls
- Supplemental\_Table\_S2.xls
- Supplemental\_Table\_S3.xls
- Supplemental\_Table\_S4.xls
- Supplemental\_Table\_S5.xls
