# Supplementary material for: TransBorrow: genome-guided transcriptome assembly by borrowing assemblies from different assemblers
Source: Genome Res. 2020 Aug;30(8):1181–90. doi: 10.1101/gr.257766.119 (PMC7462071; doi:10.1101/gr.257766.119)
Supplement: Supplemental Material [file supp_gr.257766.119_Supplemental_Material_.pdf]

Supplemental Material

TransBorrow: Genome-guided transcriptome assembly by borrowing assemblies from different assemblers

Ting Yu[1,†], Zengchao Mu[1,†], Zhaoyuan Fang[3], Xiaoping Liu[1], Xin Gao[2,*], Juntao Liu[1,*]
[1]School of Mathematics and Statistics, Shandong University (Weihai), Weihai, 264209, China
[2]Computational Bioscience Research Center (CBRC), Computer, Electrical and Mathematical Sciences and Engineering Division, King Abdullah University of Science and Technology (KAUST), Thuwal, 23955, Saudi Arabia
[3]Key Laboratory of Systems Biology, CAS Center for Excellence in Molecular Cell Science, Institute of Biochemistry and Cell Biology, Shanghai Institutes for Biological Sciences, Chinese Academy of Sciences, University of Chinese Academy of Sciences, Shanghai, 200031, China

Contents of Supplemental Material

Supplemental Notes

Supplemental Methods

Supplemental Results

Supplemental Figures (S1-S19)

Supplemental References

Supplemental Notes

Parameter setup and running commands for the mappers and assemblers

All the assemblers were tested on a server with 96 GB of RAM and a 12-core CPU. And the parameters of each genome-guided assembler were set up as their defaults with the same input file produced by HISAT2 and STAR as follows.

1) HISAT2 (version 2.0.5): HISAT2 -x Index -1 fastq1 -2 fastq2 -S SamFile --dta;

2) STAR (version 2.5.3a): STAR --outSAMstrandField intronMotif --genomeDir Index --readFilesIn fastq1 fastq2;

3) TransBorrow (version 1.2): TransBorrow -r combined.gtf -b file.bam -g genome.fa -s strandness;

4) Scallop (version 0.10.4): scallop -i file.bam -o scallop.gtf --library_type strandness;

5) StringTie (version 2.1.1): stringtie file.bam -o stringtie.gtf;

6) Cufflinks (version 2.2.1): cufflinks file.bam -o Cufflinks_Out_Dir;

7) Flux-simulator (version 1.2.1): flux-simulator –p parameters-file;

8) TACO (version 0.7.3): taco_run gtf_files.txt --filter-min-expr 0.001 -o TACO_Out_Dir;

9) StringTie-Merge (version 2.1.1): stringtie --merge stringtie2.gtf scallop.gtf cufflinks.gtf -T 0.001 -F 0.001 -o StringTie_merge.gtf;

10) Mikado (version 2.0rc4): (i) mikado configure --daijin --list list.txt --reference genome.fa --mode stringent --scoring species.yaml configuration.yaml; (ii) daijin mikado -nd configuration.yaml

Reference gene annotations used for additional real datasets

In order to evaluate the performance of the assemblers on real datasets, we downloaded the reference genome and transcriptome of the species *Homo sapiens* (version: GRCh37/hg19), *Saccharomyces cerevisiae* (version: SacCer_Apr2011/sacCer3), *Drosophila melanogaster* (version: BDGP Release 6 + ISO1 MT/dm6), *Caenorhabditis elegans* (version: WBcel235/ce11), and *Mus musculus* (version: GRCm38/mm10) from the UCSC Genome Browser at http://genome.ucsc.edu/cgi-bin/hgTables. As the UCSC Genome Browser does collect the information of the two species *Arabidopsis thaliana* and *Zea mays*, the reference genome and transcriptome of *Arabidopsis thaliana* and *Zea Mays* were downloaded from EnsemblPlants at ftp://ftp.ensemblgenomes.org/pub/plants/release-46.

Although the applications conducted in this study used the GRCh37 assembly, the users could freely choose other preferred assemblies, such as GRCh38. The genomic sequences of GRCh38 and GRCh37 are actually highly syntenic, and allows almost perfect one-to-one mapping for the majority of genomic regions, which is the basis of the widely-used UCSC liftOver utility. For the transcribed regions, GRCh38 has about 58037 annotated genes including ncRNA genes and pseudogenes (GENCODE v25), and 58028 of these genes can be perfectly mapped to the GRCh37 assembly with liftOver, at a percentage of 99.98%. In addition, we also performed a testing with GRCh38 on several data sets, which shows highly consistent results with GRCh37. Therefore, we feel it reasonable to leave the choice of genome assemblies to the users.

Supplemental Methods

Assigning edge weights of the line graphs by solving a quadratic program

The in- and out- edges (i.e. splicing junctions) for each node in splicing graph G could be accurately connected by solving a constrained quadratic program. In detail, assume that node v in splicing graph G has n in-coming edges and m out-going edges. In theory, there are $m \times n$ feasible connections between these edges. It is expected to find the true connections that the to-be-assembled transcripts pass through, based on which we designed the following programming.

$$\min \quad z = \sum_{i=1,\dots,n} (s_i - \sum_{j=1,\dots,m} w_{ij} x_{ij})^2 + \sum_{j=1,\dots,m} (c_j - \sum_{i=1,\dots,n} w_{ij} x_{ij})^2$$

$$s.t. \quad \begin{cases} x_{ij} = 1, \quad if\,(e_i, e_j) \subset P, P \in P_{\mathrm{G}} \\ \sum_{i=1,\dots,n} x_{ij} \geq 1, \quad j = 1,\dots,m \\ \sum_{j=1,\dots,m} x_{ij} \geq 1, \quad i = 1,\dots,n \\ w_{ij} \geq 0 \\ x_{ij} = \{0,1\} \\ \sum_{\substack{i=1,\dots,n \\ j=1,\dots,m}} x_{ij} = M \end{cases}$$

In the above program, s_i is the weight of the in-coming edge e_i at v, c_j the weight of the out-going edge e_j at v; x_{ij} represents a binary variable with $x_{ij} = 1$ if there is at least one transcript-representing path passing through e_i and e_j at v, and 0 otherwise, and w_{ij} represents the coverage value of all the transcript-representing paths passing through e_i and $e_{j.}$ In the objective function, $(s_j - \sum_{j=1,\dots,m} w_{ij} x_{ij})^2$ measures the deviation between the weight of the in-coming edge e_i and the sum of the weights of all the transcript-representing paths passing through e_i, and similarly for $(\mathrm{c}_j - \sum_{i=1,\dots,n} w_{ij} x_{ij})^2$. We then minimize the deviations for all the in-coming and out-going edges to find the correct connections between the in-coming and out-going edges. In the constraints, P_G is the set of extended paired-paths, and M is the minimum number of transcript-representing paths passing through node v that satisfy all the above constraints. Clearly, solving this program is NP-hard. However, it is computationally acceptable in our assembly procedure due to the specific properties of the constructed splicing graphs.

Supplemental Results

Assembly accuracy of the assemblers on additional RNA-seq samples

In addition to the evaluations on a simulated and four real data sets presented in the main text, we also evaluated the performance of all the compared assemblers on an additional 101 RNA-seq samples (see Supplemental Table S1) from the species *Homo sapiens*, *Saccharomyces cerevisiae*, *Drosophila melanogaster*, *Caenorhabditis elegans*, *Mus musculus*, *Arabidopsis thaliana*, and *Zea mays*. To evaluate the performance of the assemblers, we compared the recall and precision of the assemblers on all the data sets. At default parameters, TransBorrow showed the highest recall among all the compared assemblers on all the 101 data sets (see Figure S1 and S2), and the highest precision on 97 of the 101 data sets (see Figure S3 and S4). For all the data sets in this study, we also compared the F-score of the assemblers, computed by 2*precision*recall/(precision+recall), which is the harmonic mean of recall and precision. The higher the F-score was, the better the assembler performed. After comparison, results showed that TransBorrow showed the highest F-score on all the data sets (see Figure S10-S12). Therefore, TransBorrow performed better than all the compared assemblers on all the 101 data sets.

The main advantage of TransBorrow is to effectively assemble transcripts of those genes with complicated splicing junctions, which are usually difficult to solve. However, most reference transcripts of Saccharomyces cerevisiae are very simple. e.g., more than 95% of those reference transcripts contain only one exon. Therefore, based on our data sets, Scallop showed better performance than StringTie2 in assembling simple transcripts. When assembling complicated transcripts, StringTie2 and Scallop showed comparable performance in recall. However, The precision of StringTie2 was higher than that of Scallop in most cases.

Assembly accuracy of the assemblers on spike-in RNA-seq data

Spike-in RNA-seq data sets provided known ground truth expressed transcripts. In order to evaluate the performance of the compared assemblers by using spike-in RNA-seq data sets, we ran all the assemblers by using six spike-in RNA-seq datasets (see Supplemental Table S1) from the study (Mingfu Shao 2017) and compared their performance. After comparison, the results showed that TransBorrow demonstrated the highest recall and precision among all the applied assemblers on all the six data sets (see Figure S5 and S6 for details).

Assembly accuracy of the assemblers by using single-cell RNA-seq data

Single-cell RNA sequencing (scRNA-seq) has revolutionized traditional transcriptomic studies by extracting the transcriptome information at the resolution of a single cell. However, scRNA-seq generally brings a large amount of noise and the capture efficiency is also much

lower than traditional bulk RNA-seq. In order to evaluate the performance of TransBorrow and the other compared assemblers on scRNA-seq data sets, we ran all the assemblers on four scRNA-seq data sets (see Supplemental Table S1). For the protocol of the four scRNA-seq data sets, the first two data sets R007 and R008 used Drop-seq, Illumina Nextseq 500 protocol; the third data set R009 used BD FACSAriaIIIu Cell Sorter (BD Biosciences), Illumina HiSeq 2500 protocol; and the fourth data set R010 used Drop-seq, Illumina Nextseq 500 protocol. After comparison, we found that TransBorrow also showed the best performance over all the applied assemblers (see Figure S7 for details).

Performance of the assemblers at identifying long noncoding transcripts

Long noncoding RNAs (lncRNAs) are generally considered as non-protein coding transcripts longer than 200 nucleotides (Dinger et al. 2008; Kung et al. 2013). In order to evaluate the performance of the compared assemblers at identifying lncRNAs, we collected four human RNA-seq data sets from NCBI Sequence Read Archive (SRA) with the accession codes SRR10517380, SRR10517375, ERR2403204, and SRR10517378, which sequenced both coding and noncoding RNAs. Then we ran all the assemblers on the four data sets and compared their performance at assembling lncRNAs. Results showed that TransBorrow performed better than all the other compared assemblers at identifying lncRNAs (see Figure S8 for details).

Performance comparison between TransBorrow and Mikado

Mikado attempts to identify the most useful or best set of transcripts from multiple transcript assemblies. It tries to recover good gene models from the various options, regardless of which one has the most read support, starting from a preconception of how a gene model should look like. As demonstrated in the Mikado paper, the Mikado pipeline groups transcripts from multiple assemblies into loci and determines a representative transcript for each locus (i.e., the transcript that best fits the qualities relating to CDS, exon, intron, or UTR features) as output. Therefore, Mikado was designed for a quite different purpose from TransBorrow. Even though, we still made a comparison between the two tools on the simulated data and the four real data sets in the main text, and results showed that the recall and precision of Mikado on the simulated data was 30.57% and 42.17% based on HISAT2 mapping, respectively, which were much lower than those of TransBorrow (55.89% and 53.51%). Then the recall of Mikado on the four real data sets based on HISAT2 mapping was 9.12%, 8.91%, 10.31%, and 10.21%, versus 19.17%, 17.72%, 18.93%, and 18.13% by TransBorrow. Regarding the precision of the two tools on the four real data sets, Mikado achieved 28.1%, 28.58%, 30.26%, and 28.73%, versus 27.07%, 30.7%, 34.08%, and 32.16% by TransBorrow. For STAR mapping, the recall and precision of Mikado on the simulated data were 35.08% and 45.38%,

still much lower than those of TransBorrow (57.12% and 60.32%). Then the recall of Mikado on the four real data sets was 8.63%, 8.52%, 9.91%, and 9.68%, versus 18.02%, 16.51%, 18.01%, and 17.02% by TransBorrow. Regarding the precision of the two tools on the four real data sets, Mikado achieved 26.42%, 27.14%, 28.74%, and 26.99%, versus 26.91%, 29.77%, 32.95%, and 30.51% by TransBorrow. Its precision was slightly lower than that of TransBorrow, while its recall was much lower than that of TransBorrow.

Supplemental Figures

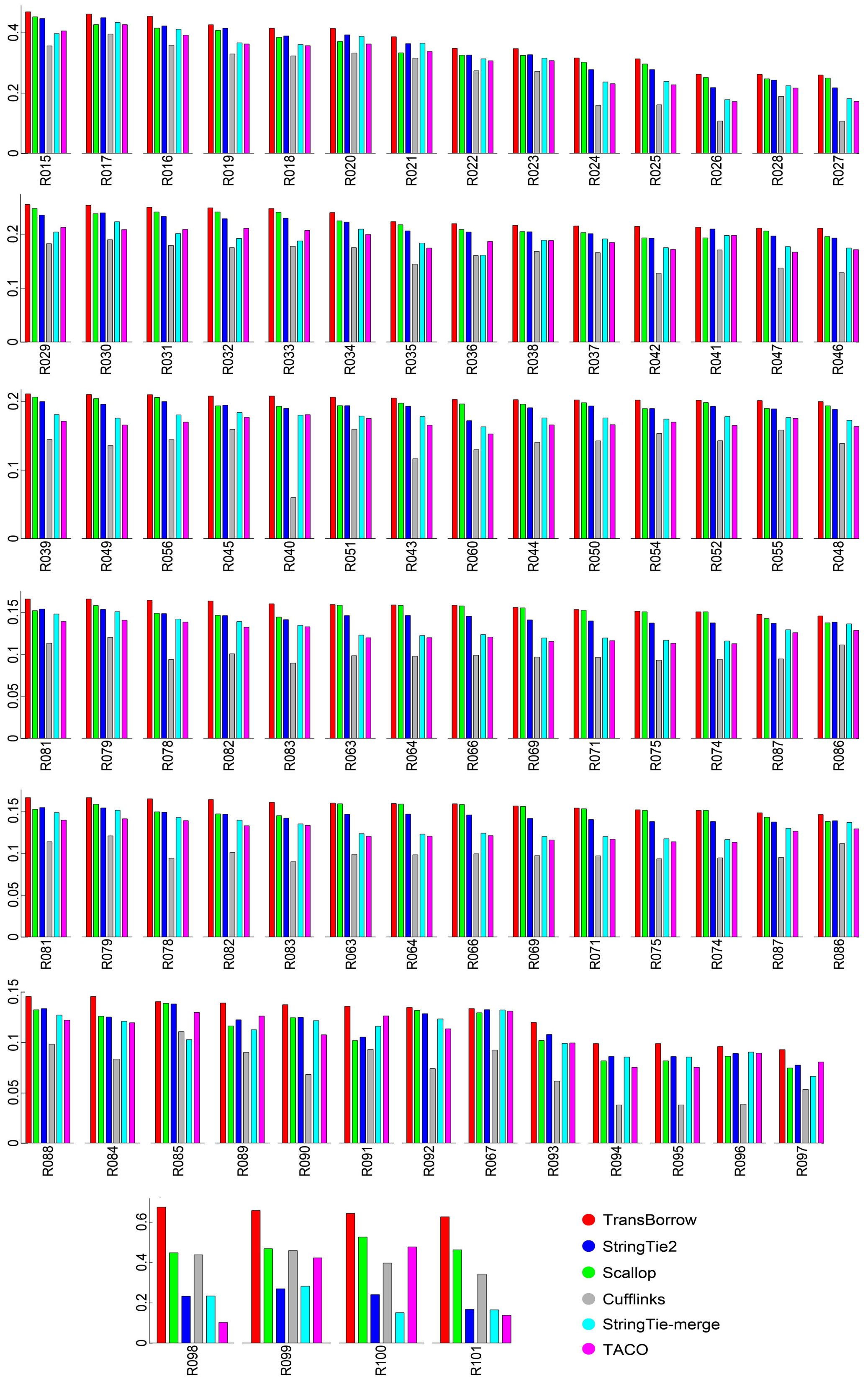

Figure S1. Recall of the assemblers on the additional RNA-seq data sets based on HISAT2 mappings.

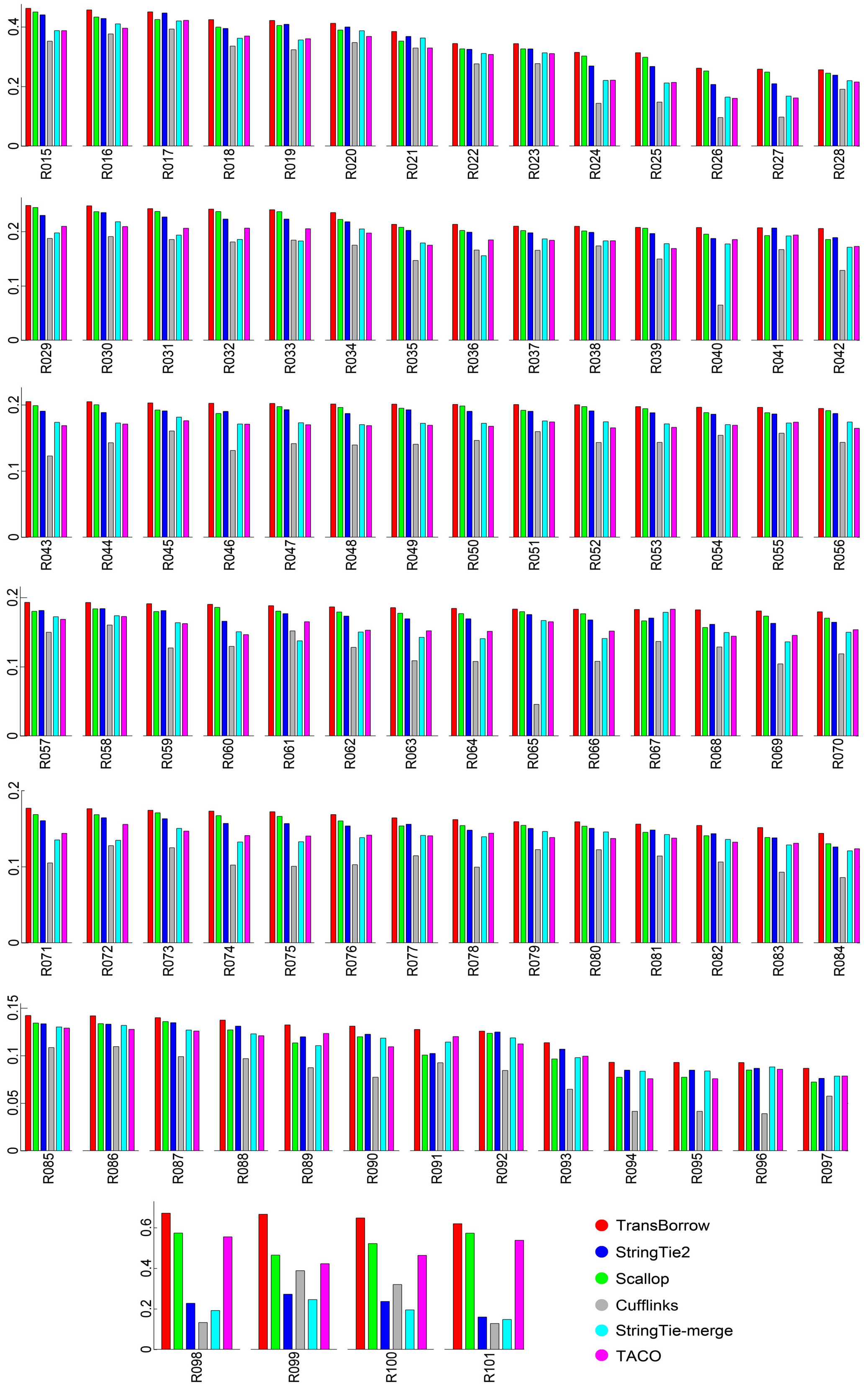

Figure S2. Recall of the assemblers on the additional RNA-seq data sets based on STAR mappings.

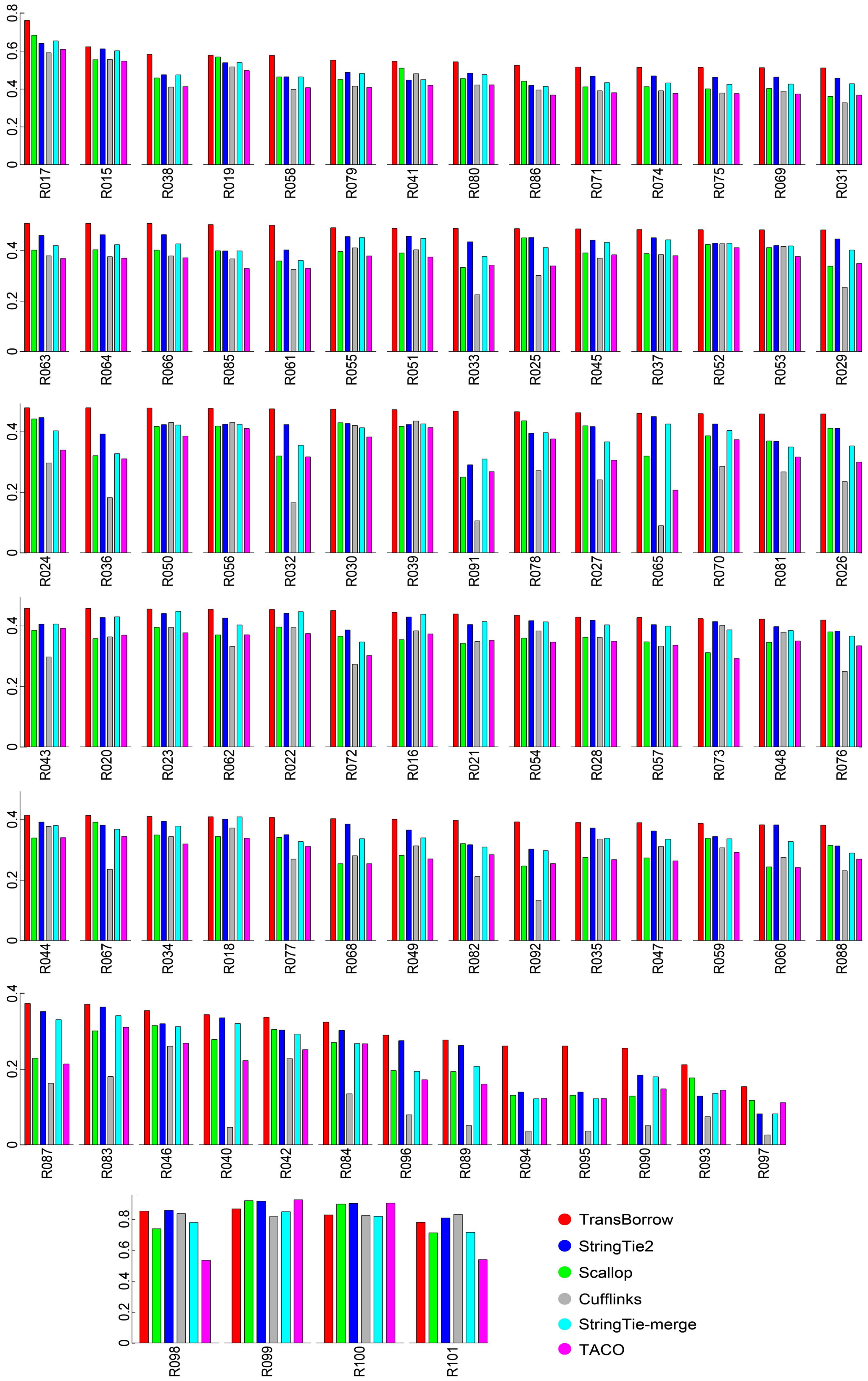

Figure S3. Precision of the assemblers on the additional RNA-seq data sets based on HISAT2 mappings.

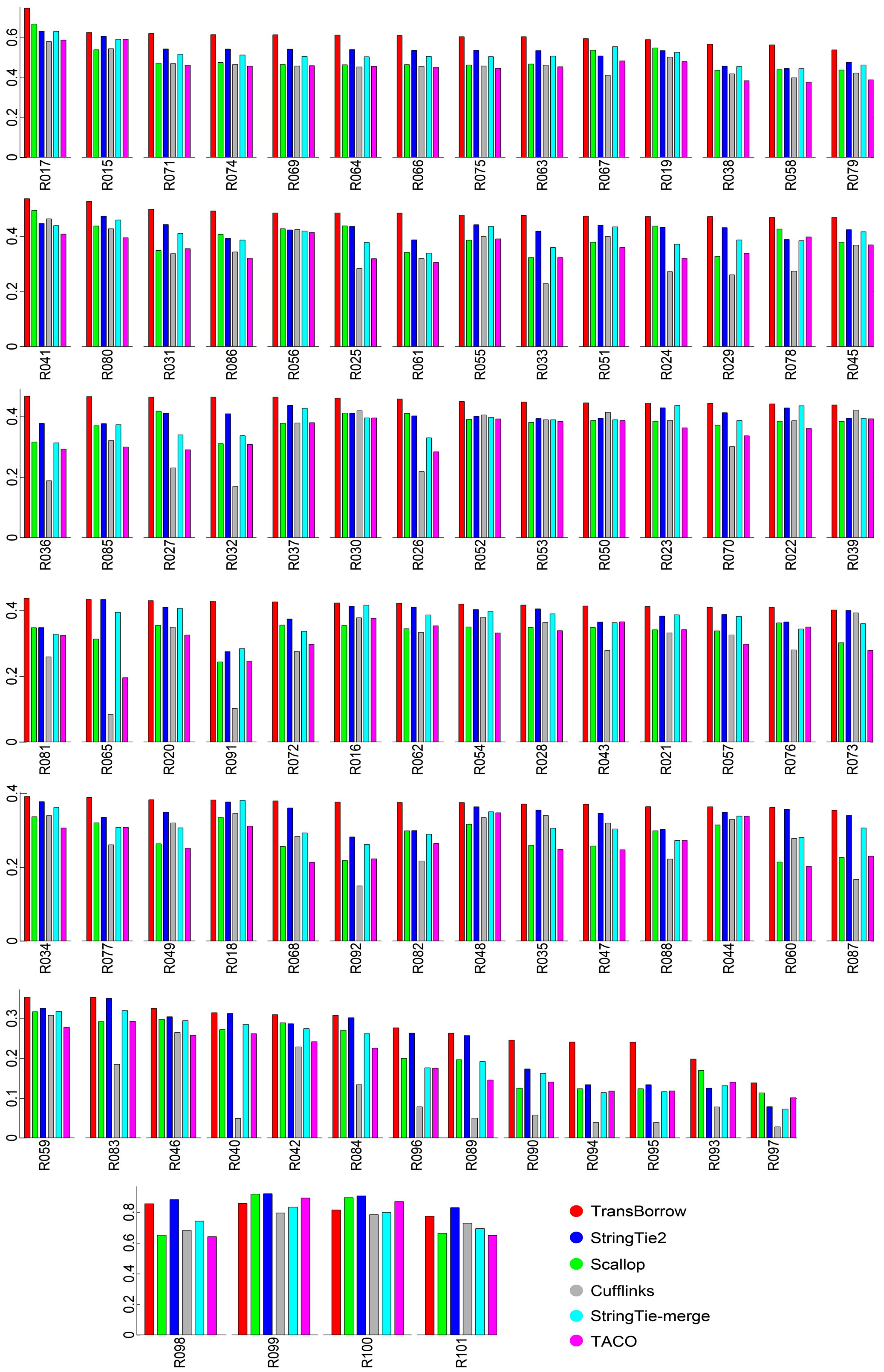

Figure S4. Precision of the assemblers on the additional RNA-seq data sets based on STAR mappings.

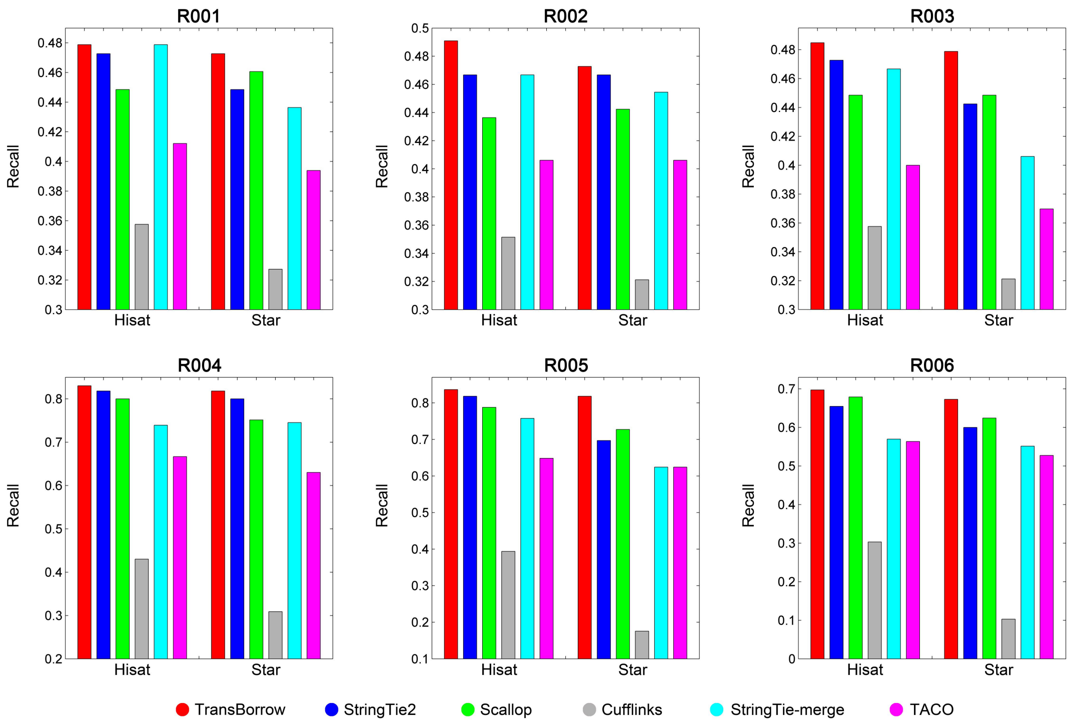

Figure S5. Recall of the assemblers on the six spike-in RNA-seq data sets.

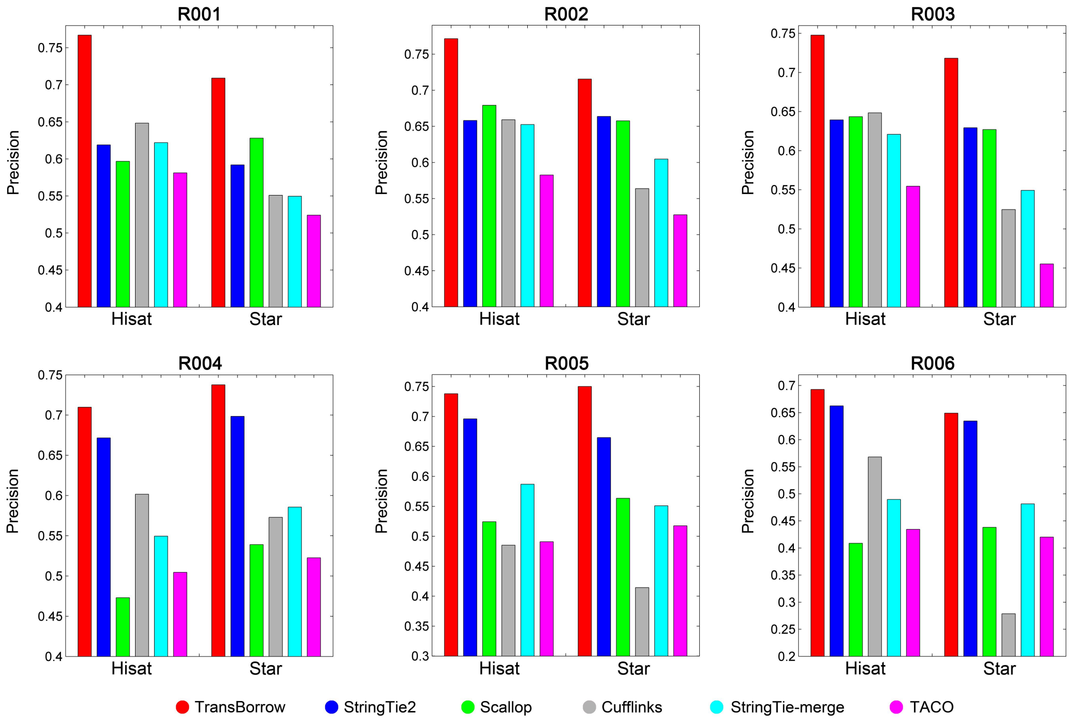

Figure S6. Precision of the assemblers on the six spike-in RNA-seq data sets.

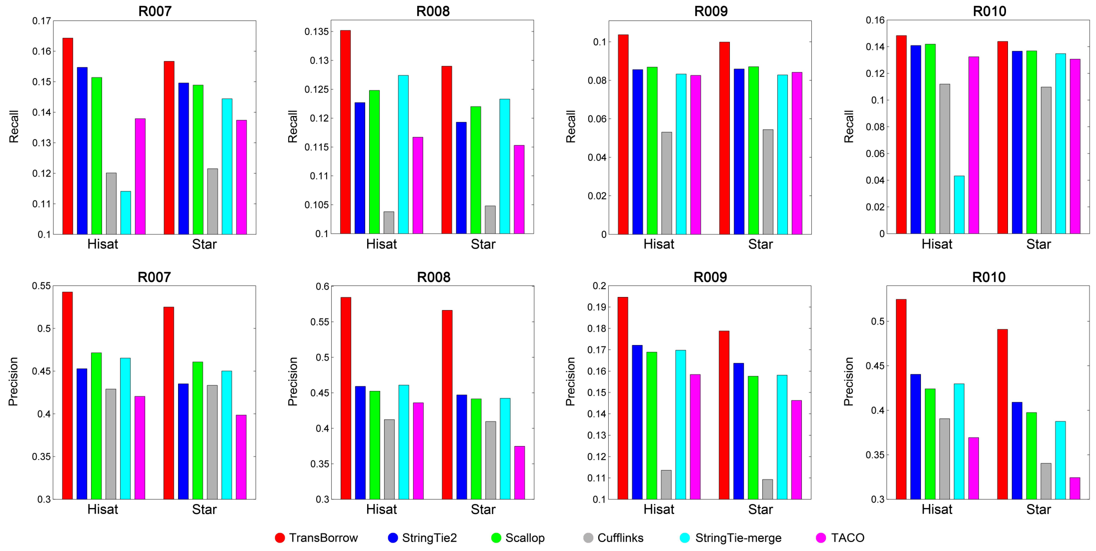

Figure S7. Accuracy comparison of the assemblers on the four single-cell RNA-seq data sets.

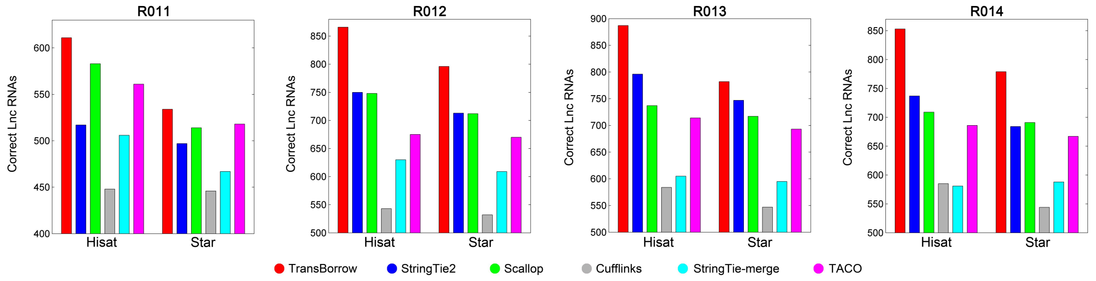

Figure S8. Correctly identified long noncoding RNAs of the assemblers on the four RNA-seq data sets, which sequenced both coding and noncoding RNAs.

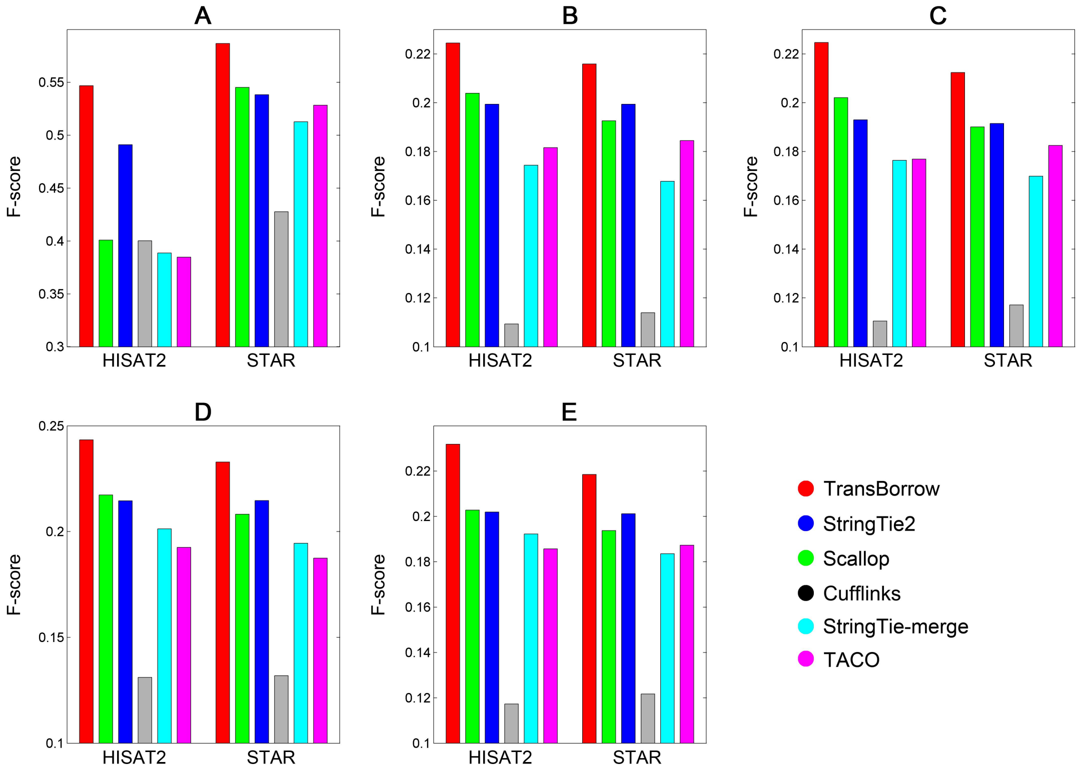

Figure S9. F-score of the assemblers on (A) simulated data set, and (B-E) real data sets R1, R2, R3, and R4.

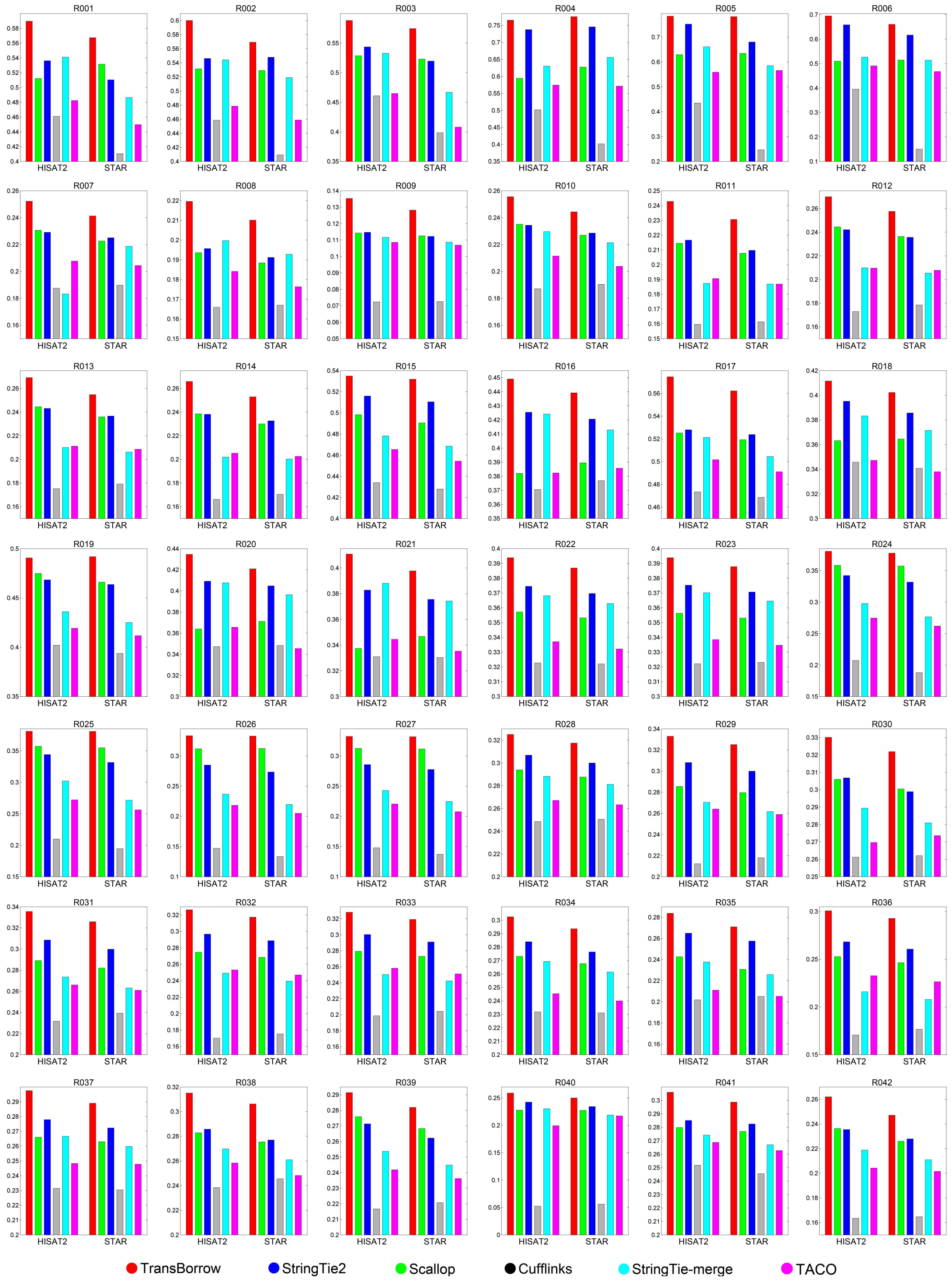

Figure S10. F-score of the assemblers on the additional RNA-seq datas from R001 to R042.

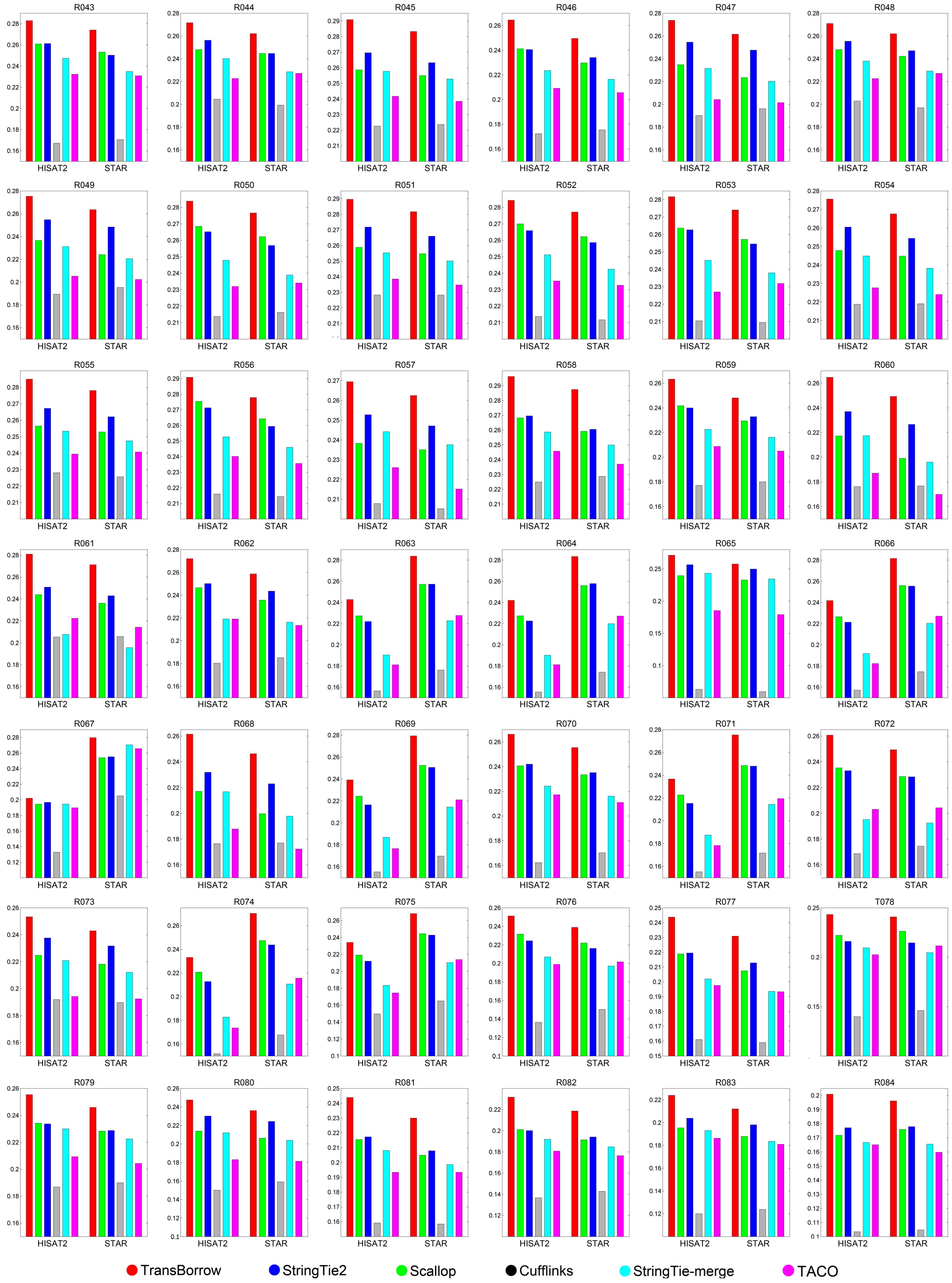

Figure S11. F-score of the assemblers on the additional RNA-seq datas from R043 to R084.

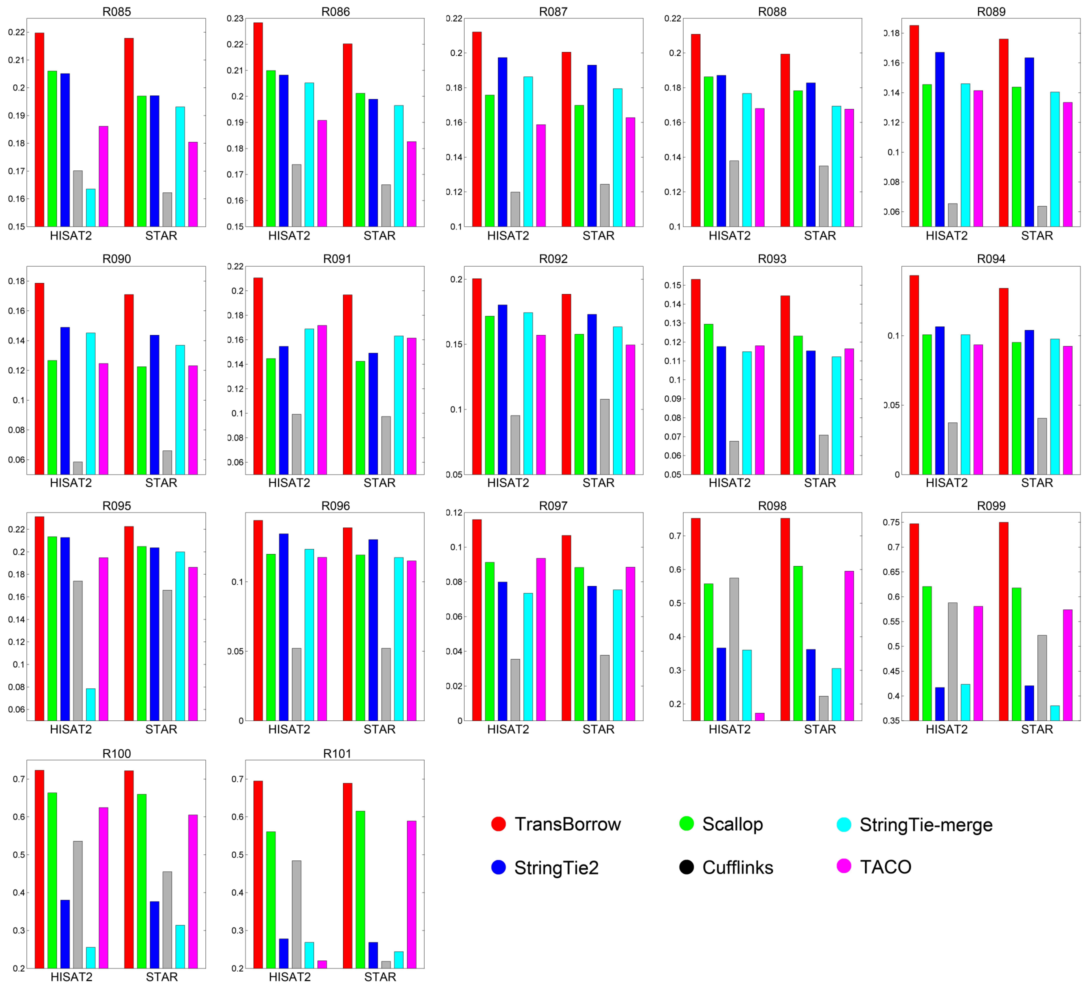

Figure S12. F-score of the assemblers on the additional RNA-seq datas from R085 to R101.

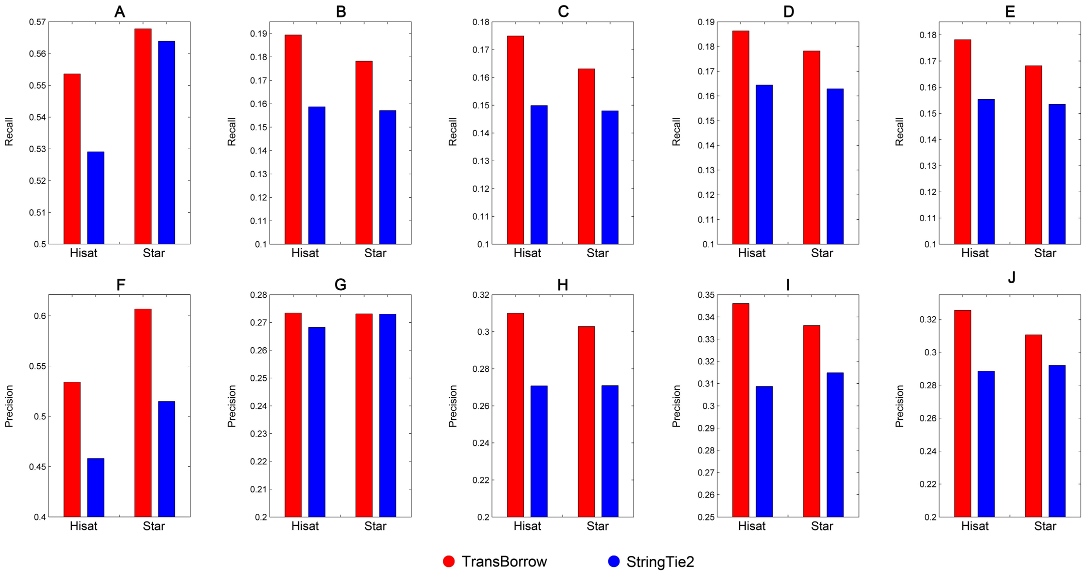

Figure S13. Accuracy comparison between TransBorrow (by using the assemblies from StringTie, Scallop, and Cufflinks) and StringTie2 in terms of recall on (A) simulated data set, and (B-E) real data

sets R1, R2, R3, and R4, and in terms of precision on (F) simulated data set, and (G-J) real data sets R1, R2, R3, and R4.

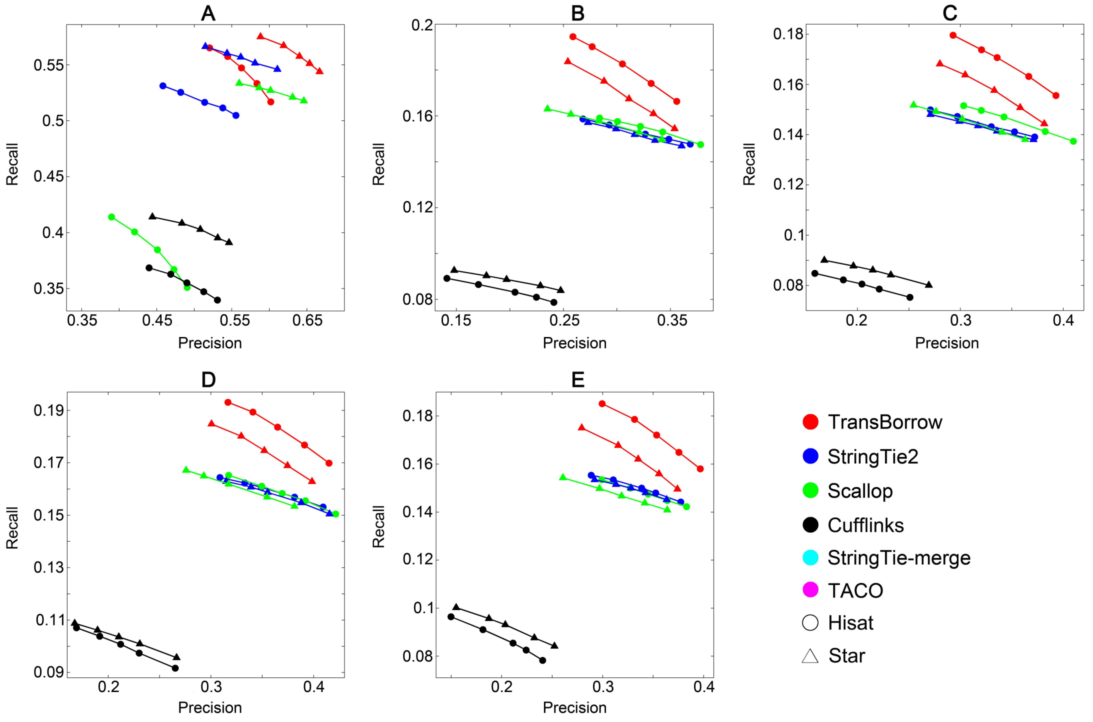

Figure S14. Recall/precision curves of the assemblers on (A) simulated data set, and (B-E) real data sets R1, R2, R3, and R4.

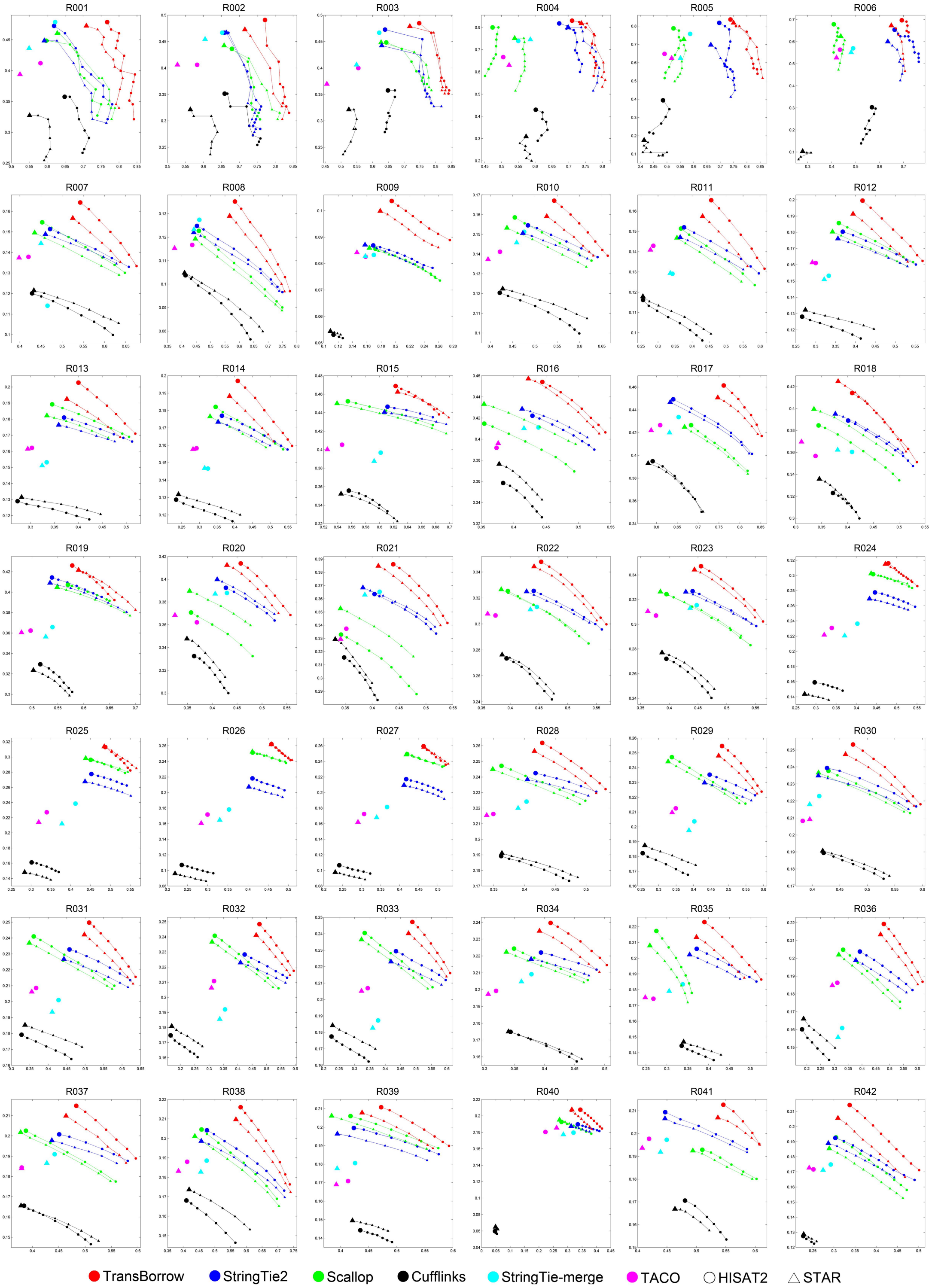

Figure S15. Recall/precision curves of the assemblers on the additional RNA-seq datas from R001 to R042, where horizontal (vertical) axis represents precision (recall).

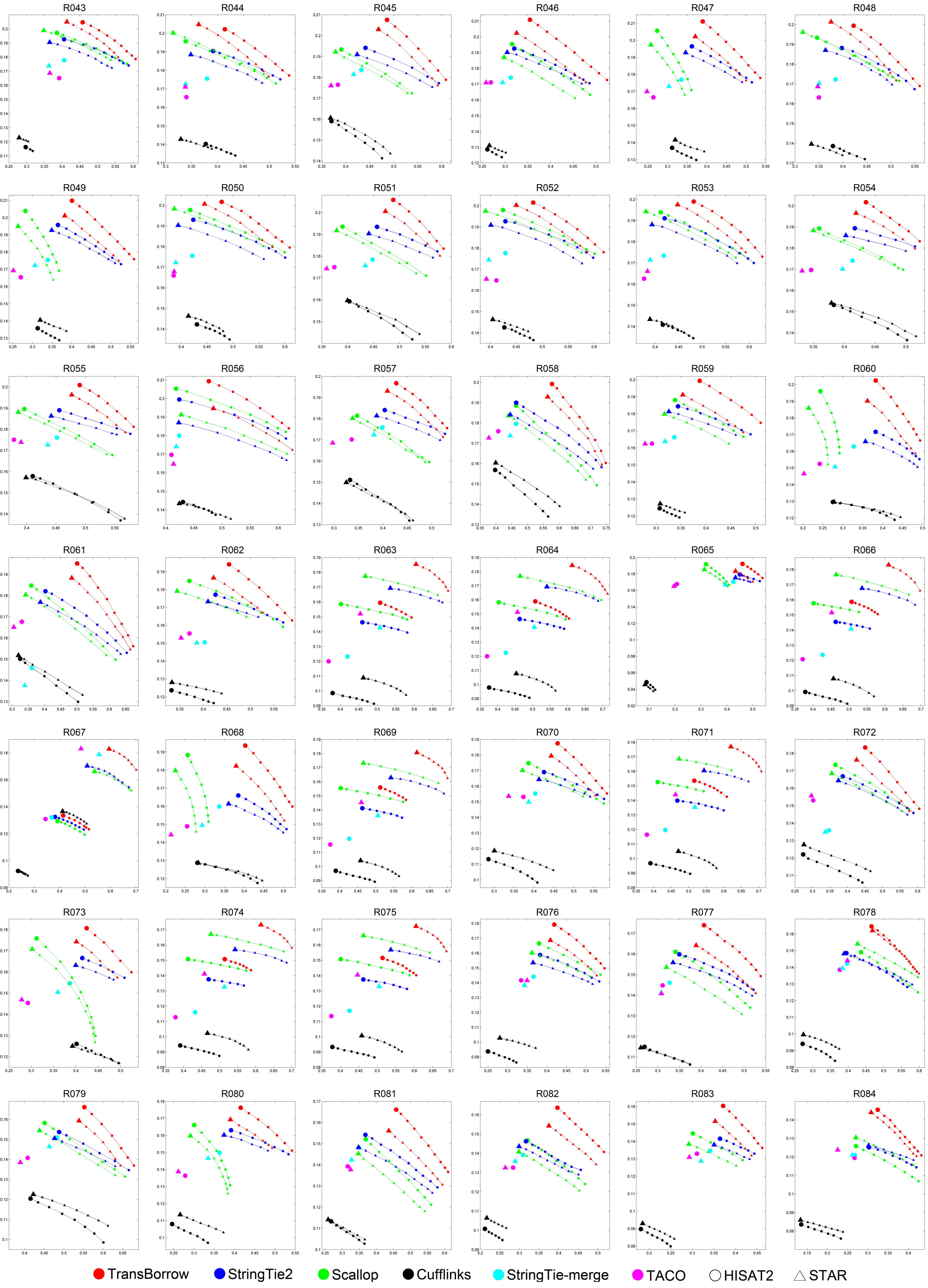

Figure S16. Recall/precision curves of the assemblers on the additional RNA-seq datas from R043 to R084, where horizontal (vertical) axis represents precision (recall).

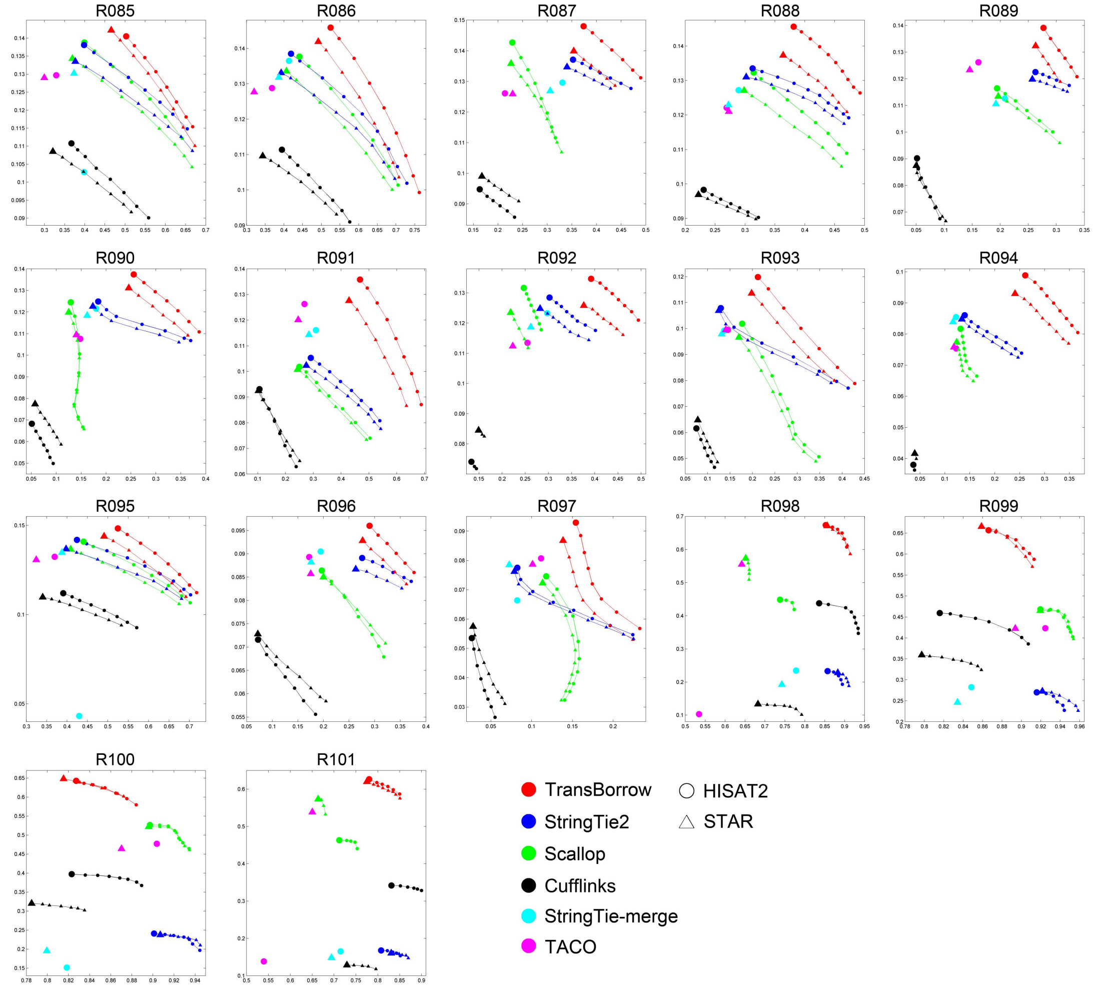

Figure S17. Recall/precision curves of the assemblers on the additional RNA-seq datas from R085 to R101, where horizontal (vertical) axis represents precision (recall).

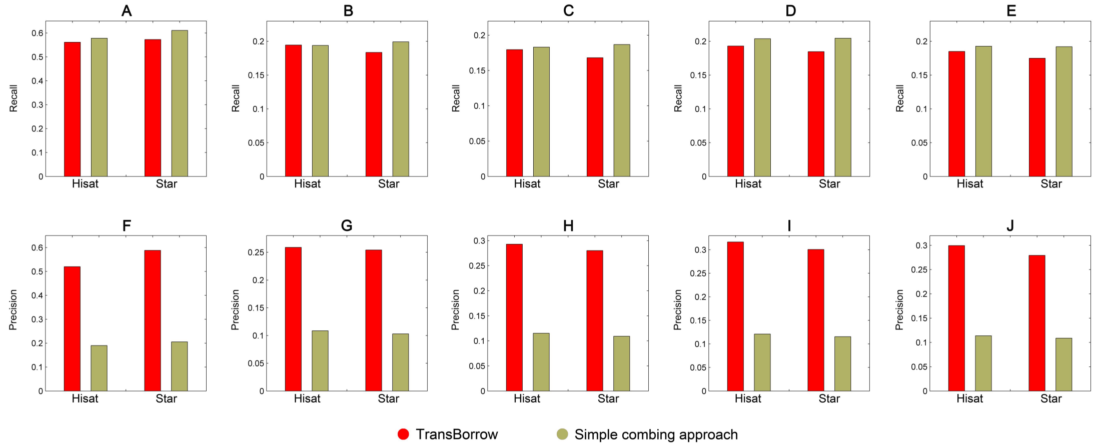

Figure S18. Accuracy comparison of TransBorrow with an approach which simply combined the assembled transcripts from different assemblers in terms of recall on (A) simulated data set, and (B-E) real data sets R1, R2, R3, and R4, and in terms of precision on (F) simulated data set, and (G-J) real data sets R1, R2, R3, and R4.

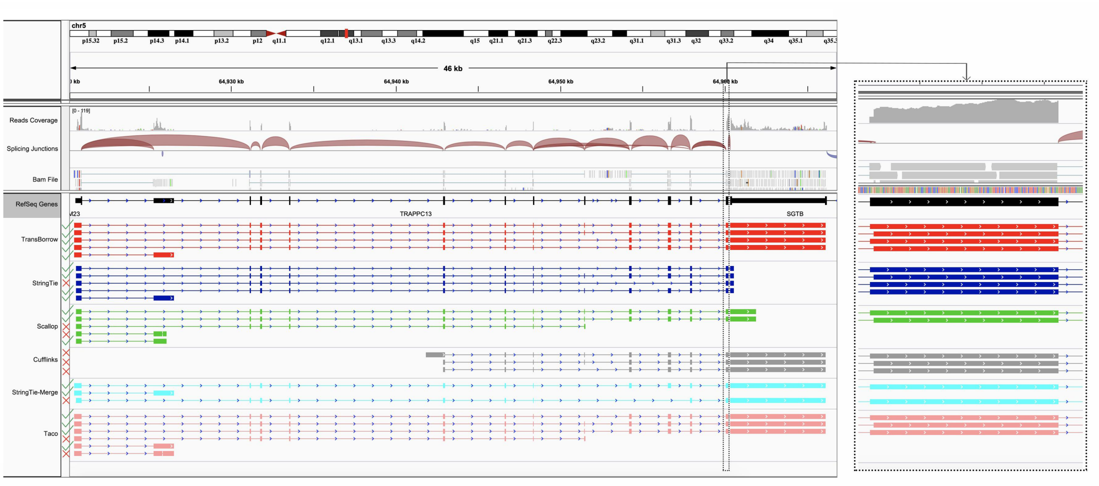

Figure S19. An IGV screenshot showing the read alignment evidence and gene structures reported by the different assemblers, where TransBorrow outperformed all the alternatives. This gene underwent complicated splicing junctions, and TransBorrow correctly assembled all the five transcripts. However, one transcript was erroneously predicted by StringTie2, and two by Scallop. Cufflinks only assembled three transcripts and all of them were false positives. StringTie-merge also predicted three transcripts and one of them was erroneously assembled. TACO output six transcripts and only four of them were correctly assembled.

Supplemental References

Dinger ME, Amaral PP, Mercer TR, Pang KC, Bruce SJ, Gardiner BB, Askarian-Amiri ME, Ru K, Solda G, Simons C et al. 2008. Long noncoding RNAs in mouse embryonic stem cell pluripotency and differentiation. *Genome research* **18**(9): 1433-1445.

Kung JT, Colognori D, Lee JT. 2013. Long noncoding RNAs: past, present, and future. *Genetics* **193**(3): 651-669.

Mingfu Shao CK. 2017. Accurate assembly of transcripts through phase-preserving graph decomposition. Nature biotechnology 35: 1167 - 1169.
